# Evolution of Resistance to Avian Malaria Infection in an Endemic Hawaiian Honeycreeper

**DOI:** 10.1002/ece3.73550

**Published:** 2026-05-08

**Authors:** John H. Neddermeyer, Loren Cassin‐Sackett, Eben H. Paxton, Michael G. Campana, Robert C. Fleischer, Jeffrey T. Foster

**Affiliations:** ^1^ Pathogen and Microbiome Institute Northern Arizona University Flagstaff Arizona USA; ^2^ Department of Biology University of Louisiana Lafayette Louisiana USA; ^3^ Center for Conservation Genomics Smithsonian National Zoo and Conservation Biology Institute Washington DC USA; ^4^ U.S. Geological Survey Pacific Island Ecosystems Research Center Hawaii National Park Hawaii USA

**Keywords:** conservation, disease ecology, evolution of resistance, rapid evolution, wildlife infectious disease

## Abstract

Malaria parasites in the genus *Plasmodium* are ubiquitous infectious agents. Most avian species effectively manage infection and experience limited population effects, with a notable exception in the Hawaiian Islands. Since Hawaiian honeycreepers evolved over ~7 million years without blood parasites, the introduction of *Plasmodium relictum* had devastating consequences—including widespread extinctions and extirpations. However, honeycreeper species have been differentially affected by malaria. Using whole genome sequences for 35 Oʻahu ʻamakihi ( *Chlorodrepanis flava* ), we conducted a genome‐wide association study to identify single nucleotide polymorphisms (SNPs) correlated with avian malaria infection. Infection status was determined using real‐time PCR on blood samples. We identified 582 SNPs associated with malaria infection. Annotations included long ncRNAs, genes associated with transcription regulation, apoptosis, T‐cell and complement activation, autophagosome assembly and transport, and intracellular calcium flux. By identifying genes regulating intracellular calcium ions, a key signaling molecule in *Plasmodium* replication, our work shows that host limitations on parasite replication may not solely depend on the adaptive immune response. Host responses can also involve restricting intracellular components essential for completion of parasite life cycles. This adds to a growing wildlife disease literature indicating a broad range of mechanisms of disease resistance rather than common pathways.

## Graphical abstract

Hawaiian honeycreepers, a group of endemic song birds, represent one of the most stunning examples of an adaptive radiation. An evolutionary history devoid of malaria‐causing parasites has made them incredibly susceptible to disease. This work sought to identify genomic correlates of avian malaria infection.

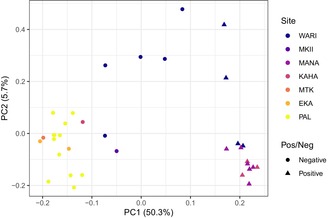

## 1 Introduction

Infectious disease has been one of the most constant selective pressures in the evolution of birds and mammals (Enard et al. 2016; Quach et al. 2016; Shultz and Sackton 2019). Hosts often evolve responses to infectious agents, and host genetics are frequently identified as a major component of infection outcomes in both humans (Augusto et al. 2023; Band et al. 2015; Kirkpatrick et al. 2022) and wildlife (C. Armstrong et al. 2018, 2019; Cassin‐Sackett et al. 2019; Epstein et al. 2016; K. L. Paxton et al. 2023). The rapid emergence of novel infectious agents has both presented heightened selective pressures and imperiled wildlife, with devastating consequences for biodiversity (Azat et al. 2022; Daszak et al. 2000; Hoyt et al. 2021). It is unknown whether evolutionary responses to novel parasites occur primarily in immune genes or genes involved in regulating parasites via other mechanisms. Activation of the immune system in response to infection can have fitness costs; therefore, in certain circumstances, it may be beneficial for hosts to manage infection through non‐immune‐mediated mechanisms (Lilley et al. 2019).

Malarial disease is caused by vector‐borne intracellular parasites in the haemosporidian genus *Plasmodium* that have two main life‐history stages in avian hosts: during initial infection, the parasite infects host organs, and after undergoing replication, egresses and infects host red blood cells (C. T. Atkinson 2008). Avian *Plasmodium* are known to go through multiple cycles of red blood cell and tissue phases of infection in the same individual (Valkiūnas and Iezhova 2017). Avian malaria infection does not often result in massive population die‐offs (Fecchio et al. 2021; Valkiunas et al. 2018). Although there have been documented population declines attributed to *Plasmodium* infection (Dadam et al. 2019), evidence suggests there is a fitness cost to maintaining a chronic infection (Asghar et al. 2015; Schoenle et al. 2017).

Although avian malaria can be found on every continent except Antarctica, the parasite is believed to have not reached the Hawaiian Islands until the turn of the 20th century (Beadell et al. 2006; Warner 1968). Its introduction is a preeminent example of the severe consequences of introduced infectious disease on native species. Malaria in Hawaiʻi, caused by *Plasmodium relictum* strain GRW4, appears to have been the main driver of honeycreeper extinctions (C. T. Atkinson 2023; Kyriazis et al. 2025). Challenge experiments found that from a single infectious mosquito bite, Hawaiʻi ʻamakihi (*Chlorodrepanis v. virens*), ʻapapane ( *Himatione sanguinea* ), and ʻiʻiwi (*Drepanis coccinea*) had mortality rates of 65%, 62.5%, and 90%, respectively (C. T. Atkinson et al. 1995, 2000; Yorinks and Atkinson 2000). The severe mortality observed in honeycreepers is likely the result of an evolutionary history devoid of malaria‐causing parasites. Due to the isolation of the Hawaiian Islands, for ~5.8–7.2 million years of honeycreeper evolution (Freed et al. 1987; Lerner et al. 2011), malaria was likely absent. Only after the introduction of mosquitoes in the early 19th century and malaria‐infected non‐native bird species was sustained transmission possible (C. T. Atkinson and LaPointe 2009; Foster 2009). This confluence of events makes avian malaria in Hawaiʻi a useful system for understanding evolutionary responses to recent selective pressures of an introduced disease.

In the Hawaiian Islands, malaria often limits honeycreepers to high elevation areas that until recently had been refuges from infection (C. T. Atkinson et al. 2005; C. T. Atkinson and Samuel 2010; Samuel et al. 2015; van Riper et al. 1986; Warner 1968). Thermal limits on parasite development in mosquito vectors reduce transmission, with temperatures below 21°C drastically slowing parasite development (LaPointe et al. 2010). However, in recent years, malaria has expanded into increasingly higher elevations (C. T. Atkinson et al. 2014), and low elevation islands likely lack disease‐free refugia entirely (Neddermeyer et al. 2023). As the climate continues to warm, the expansion of avian malaria to higher elevations in the Hawaiian Islands is a constant threat to endemic honeycreepers (Benning et al. 2002; Hobbelen et al. 2012; Kyriazis et al. 2025; Liao et al. 2017), highlighting the importance of identifying protective alleles.

Experimental data clearly indicate reduced survival in populations of high‐elevation Hawaiʻi ʻamakihi (*C. v. virens*) with low avian malaria exposure versus relatively high survival in low‐elevation populations that historically faced and currently face high exposure to malaria (C. T. Atkinson et al. 2013). Hawaiʻi ʻamakihi demonstrate population structure, particularly between high and low elevation populations (Eggert et al. 2008; Foster et al. 2007), which has likely resulted in population‐specific survival strategies for malaria infection (Cassin‐Sackett et al. 2019). Despite mounting evidence that Hawaiʻi ʻamakihi have evolved infection survival mechanisms to *P. relictum* in areas of high malaria prevalence (Atkinson et al. 2025, *in revision*; Paxton et al. 2023), limited work has been done on Oʻahu ʻamakihi ( *Chlorodrepanis flava* ), a sister taxon. Data from an unpublished study suggest some evidence of population structure, and that when Oʻahu ʻamakihi are challenged with avian malaria, there is 100% survival (Krend 2011). Avian malaria is present year‐round across the island of Oʻahu, with no infection‐free refugia, and no difference in infection prevalence between juvenile and adult birds. This suggests birds are exposed to malaria shortly after hatching (Neddermeyer et al. 2023).

Whole‐genome association studies in the Hawaiian honeycreeper malaria system are lacking. Although mutations occurring within protein‐coding genes are important, gene regulatory networks can be hotspots of adaptive evolution (Peter and Davidson 2011; Roscito et al. 2018). Sequencing the whole genome enables identification of infection‐associated genetic changes not just within protein‐coding regions, but within gene regulatory regions. Studies published thus far have focused on reduced representation sequencing approaches, such as transcriptomics and target‐capture‐seq (Cassin‐Sackett et al. 2019, 2025; K. L. Paxton et al. 2023). Our goal was to identify alleles associated with malaria infection in Oʻahu ʻamakihi, thereby identifying resistance mechanisms. Given that avian malaria is ubiquitous on Oʻahu and the potential fitness costs of harboring malaria infection, we hypothesized that infection‐associated variants would be found within genes that control parasite replication, limiting blood‐borne parasite prevalence.

## 2 Materials and Methods

### 2.1 Sample Collection, Sequencing, and Parasite Molecular Detection

**FIGURE 1:**
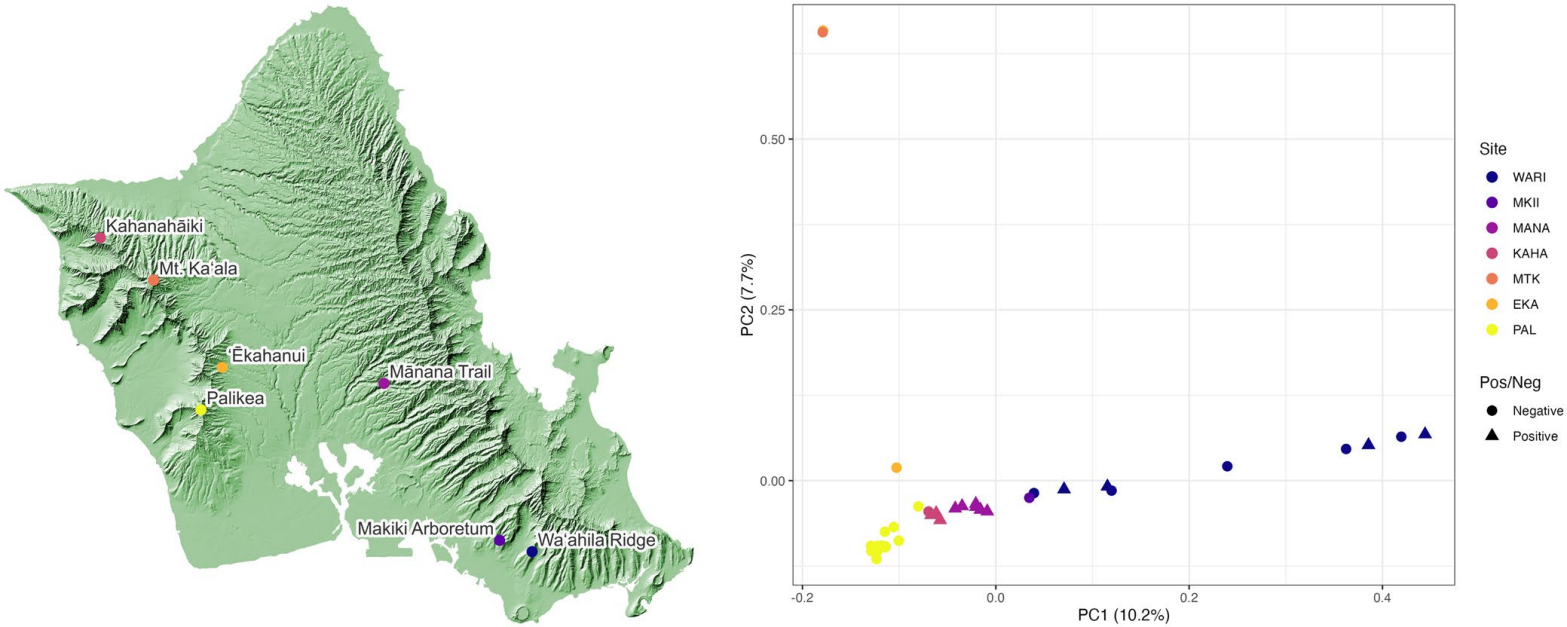
Oʻahu ʻamakihi sampling locations and principal component analysis (PCA) on 426,241 single nucleotide polymorphisms (SNPs), after pruning SNPs with *r* ≥ 0.1, with 20 Principal Component axes evaluated. PCA points represent sampled individuals, and PCA site colors correspond to the map of Oʻahu. WARI = Waʻahila Ridge, MKII = Makiki, MANA = Mānana Trail, KAHA = Kahanahāiki, MTK = Mt. Kaʻala, EKA = ʻĒkahanui, PAL = Palikea. Circles and triangles symbolize if a sample was PCR negative or positive for avian malaria.

In March and May 2021, 35 Oʻahu ʻamakihi blood samples were collected across five sites on the island of Oʻahu (E. H. Paxton 2022), with three additional blood samples collected in 2015 and 2016 from two additional sites (Neddermeyer et al. 2023; Figure 1; Table S1). Sites were chosen to optimize the number of Oʻahu ʻamakihi sampled. All collected samples were authorized under the U.S. Geological Survey (USGS) Bird Banding Lab (23064), Island of Hawai‘i Protected Wildlife Permit (WL19‐20), and the University of Hawaiʻi's Animal Care and Use Committee (protocol # 18–2916). We extracted genomic DNA from avian blood samples using the Qiagen DNeasy Blood & Tissue kit (Qiagen, Hilden, Germany) following the manufacturer's protocol for the Purification of Total DNA from Animal Blood for cells with nucleated erythrocytes.

Prevalence of *P. relictum* infection was determined using real‐time PCR following protocols outlined in Neddermeyer et al. (2023) and Paxton et al. (2023). We considered a sample positive for *P. relictum* infection if the sample crossed the cycle threshold within 40 cycles.

One Oʻahu ʻamakihi sample (band #2631–31,193) was processed using 10X Genomics linked‐read protocols to generate a high‐quality reference genome. Briefly, a linked‐read library was prepared using the Chromium Reagent Kit following the manufacturer's protocol (10X Genomics 2018). High molecular weight DNA was extracted and barcoded before being sheared for 150 bp paired‐end sequencing on an Illumina HiSeq. The genome for this sample was *de novo* assembled using 10X Genomics Supernova Assembler v2.1.1 software (Weisenfeld et al. 2017). Raw 10X linked‐read data were used as input into Supernova without any read cleaning, with Supernova output format set to “pseudohap”.

Expected gene content was evaluated using Benchmarking Universal Single Copy Orthologs, BUSCO v5.0 (Manni et al. 2021) and the Aves lineage database aves_odb10. BUSCO scanned the genome for the presence of 8338 highly conserved avian orthologs. The genome for sample 2631‐31193 was the reference genome used in all analyses.

Genomic DNA library construction for whole‐genome sequencing (WGS) was performed for the remaining samples using the KAPA Hyper Prep Kits for Illumina NGS platforms and KAPA UDI Primer Mixes per manufacturer's protocol, with double‐sided size‐selection performed after sonication. Final libraries were quantified on an Applied Biosystems QuantStudio 7 Flex Real‐Time PCR System using the KAPA SYBR FAST ROX Low qPCR Master Mix for Illumina platforms. The libraries were pooled together at equimolar concentrations, and quality was assessed with the Agilent Technologies TapeStation 4150 System. Final quantification by qPCR preceded sequencing of the final library. Samples were sequenced on the Illumina NextSeq1000 to 15X coverage using the NextSeq1000/2000 P2 600 Cycle Reagent Kit using the standard Illumina protocol.

### 2.2 Sequence Processing and SNP Detection

To include the genome of sample 2631‐31193 in our population genomic analyses, we generated a BAM file for the 10X‐linked reads using 10X Genomics LongRanger software tool (Marks et al. 2019) by aligning raw sequence reads back to the assembled reference genome. For the remaining samples, FastQ reads were trimmed for Illumina adapter sequences and sequence quality using Trimmomatic 0.39 (Bolger et al. 2014) with the following parameters: ILLUMINACLIP:adapters.fa:2:30:5 LEADING:20 TRAILING:20 SLIDINGWINDOW:4:20 MINLEN:36. Trimmed FastQ reads were aligned to the reference Oʻahu ʻamakihi genome using BWA‐MEM 0.7.17 (Li and Durbin 2010). Reads were filtered using SAMtools 1.11 (Li et al. 2009) to only include paired‐end reads that were mapped in the proper orientation, and within an expected distance of one another based on insert size. Picard MarkDuplicates 2.24.1 (http://broadinstitute.github.io/picard/) was used to mark and remove PCR duplicates. Reads were realigned around indels using Genome Analysis ToolKit (GATK) 3.8–1‐0‐gf15c1c3ef IndelRealigner (Van der Auwera and O'Connor 2020). We removed two samples that had a mean sequencing depth < 4× and percent reference genome coverage < 85%.

To identify outlier samples, we conducted a PCA using PCAngsd v1.36.3 (Meisner and Albrechtsen 2018) and visualized sample clustering. We generated a genotype likelihood file using ANGSD version 0.941–22‐gc877e7f (Korneliussen et al. 2014) with parameters ‐doMajorMinor 1 ‐SNP_pval 1e‐6 ‐doMaf 1 ‐minMapQ 20 ‐minQ 20. The genotype likelihood file was then provided to PCAngsd with option ‐iter 1000. Based on sample clustering, this analysis identified three problematic samples, 2591–08576, 2591–08542, and 2591–08536 (Figure S1). To check for blood sample contamination, we conducted a competitive alignment where we concatenated red‐billed leiothrix ( *Leiothrix lutea* ; GCA_013400445.1_ASM1340044v1_genomic.fna) and warbling white‐eye ( *Zosterops japonicus* ; GCA_017612475.1_CCG_JAWE_1.0_genomic.fna) genomes onto our Oʻahu ʻamakihi reference genome and reran our alignment pipeline for the three outliers (Feuerborn et al. 2020). Warbling white‐eye and red‐billed leiothrix were the two most common sampled bird species across all sites, and blood samples from these species were collected at the same banding station as blood collection from Oʻahu ʻamakihi. After competitive alignment, we determined 2591–08576 was likely not collected from an Oʻahu ʻamakihi, a possible mislabeling error. Samples 2591–08542 and 2591–08536 were likely contaminated with blood from red‐billed leiothrix or warbling white‐eye. However, after filtering non‐Oʻahu ʻamakihi reads, samples retained enough sequence information to meet sequence depth and breadth requirements and were retained in downstream analyses. After filtering contaminating reads, samples 2591–08542 and 2591–08536 were no longer outliers based on PCA (Figure S2).

We identified and removed reads that aligned to the Oʻahu ʻamakihi Z sex chromosome. Putative sex chromosomes were identified using a reference zebra finch ( *Taeniopygia guttata* ) Z chromosome downloaded from Ensembl and tools in the package mummer4.0.0beta2 (Kurtz et al. 2004). Nucmer was run using the parameters ‐c 200 ‐b 200. The output delta file was then filtered to only include scaffolds that had an alignment length > 1000 bp and sequence identity > 92%. The reference zebra finch Z chromosome was 75 MB in length; 67 MB of our Oʻahu ʻamakihi reference genome aligned with the zebra finch Z chromosome. Scaffolds identified as Z chromosome were removed from sample BAM files for subsequent analyses.

Variants were called using BCFtools 1.20 mpileup and BCFtools 1.20 call functions (Danecek et al. 2021). A list of sample BAM files was input into BCFtools mpileup and BCFtools call was set to ‐multiallelic‐caller. Bases with quality scores and alignments with mapQ scores < 20 were omitted, and max sequence depth was set to 100×. After variant calling, we filtered our VCF file to remove variants with RPBZ statistic < 0.1, allele count (AC) < 2, and number of sequences supporting indels (IDV) ≤ 3.

For our analysis of Oʻahu ʻamakihi population structure and infection association analyses, we filtered variants using VCFtools v0.1.17 (Danecek et al. 2011) to only include biallelic sites, positions that were genotyped for all samples, and sites with a minor allele frequency (MAF) of 10%. At a MAF of 10%, we ensured that at least three samples at a given position contained the allele being evaluated.

### 2.3 Assessing Population Structure and Genome‐Wide Heterozygosity

We used our unpruned SNP set to count the number of heterozygous sites per sample and then divided the number of heterozygous sites by the total number of sites that had been genotyped to estimate genome‐wide heterozygosity. We used a Wilcoxon Rank Sum Test to determine if genome‐wide heterozygosity was different between infected and uninfected individuals. To evaluate population structure, we pruned SNPs with an *r* ^*2*^ > 0.1 for linkage‐disequilibrium (LD) using PLINK v1.90b7.2 (Chang et al. 2015) with parameters ‐indep‐pairwise 50 10 0.1. Using pruned SNPs, we evaluated population structure using ADMIXTURE v1.3.0 (Alexander et al. 2009) and a principal component analysis (PCA) in PLINK. We evaluated ADMIXTURE *K*‐values from 2 to 10 using 5‐fold cross‐validation. ADMIXTURE results were visualized spatially using R package *mapmixture* (Jenkins 2024).

### 2.4 Identifying SNP Associations

We took two approaches to identifying SNPs associated with avian malaria infection based on our LD‐pruned SNP set. Using VCFtools, for each SNP, we calculated pair‐wise F_ST_ values between infected and uninfected samples. We calculated 95% bias‐corrected and accelerated (BCA) confidence intervals using the function bca in R package *coxed* v0.3.3 (Kropko and Harden 2020); if a SNP had an F_ST_ value above the upper 95% BCA CI it was identified as associated with avian malaria infection. To ensure high F_ST_ value SNPs were not the result of incidental *P. relictum* genomic DNA sequencing, we extracted flanking sequences 400 bp and 4000 bp up and downstream from a subset of our most differentiated SNPs and used BLASTn to identify sequence hits to a *P. relictum* genome downloaded from NCBI, GCF_900005765.1. Our BLASTn results did not identify any significant hits to the *P. relictum* genome, indicating high F_ST_ value SNPs were not the result of incidental *P. relictum* genome sequencing.

We then conducted a genome‐wide association study (GWAS) using Linear Mixed Model (LMM) and Linear Model (LM) frameworks in the software package GEMMA v0.98 (Zhou and Stephens 2014). When implemented as an LMM, GEMMA allows for testing associations between SNPs and phenotypes while controlling for population structure using a supplied sample relatedness matrix. In both LMM and LM frameworks, *p‐*values were calculated for each SNP using a likelihood ratio test. GEMMA fits the binary phenotype as a linear model to improve computation efficiency and robustly reduce genomic inflation factor by controlling population structure. As a result, beta coefficients should be interpreted as a change in probability, not an odds ratio (Zhou and Stephens 2014). Q–Q plots and genomic inflation factor were assessed for both model formulations to identify violations. To account for multiple testing, we used a Bonferroni correction, with a significance threshold of *p*‐value ≤ 1.17 × 10^−7^, and a *q‐value* threshold of 0.30. Two types of significance thresholds ensured we captured SNPs that could prove significant with larger sample sizes. Due to small sample size, we were unable to model sample site as a covariate in either the LM or LMM framework. We attempted to further control for population structure by iteratively adding principal components 1–5 from our linkage‐pruned SNP set.

### 2.5 Annotating SNPs Associated With Infection

We constructed a multispecies whole genome alignment using Cactus v2.82 (J. Armstrong et al. 2020). The alignment contained 19 Hawaiian honeycreeper genomes (Campana et al., n.d. *In prep*; Neddermeyer et al., n.d. *In prep*), and zebra finch assembly bTaeGut1_v1.p downloaded from Ensembl. The guide tree was constructed using a coalescent algorithm implemented in wASTRAL v1.15.2.3 with mode Weighted ASTRAL–Hybrid (Zhang and Mirarab 2022), using extracted BUSCO gene trees as input. We then used halLiftover (Hickey et al. 2013) to annotate the Oʻahu ʻamakihi reference genome with zebra finch annotations. The zebra finch bed file used as input was filtered to only include Ensembl canonical transcripts. As a part of preprocessing for the multispecies alignment, the Oʻahu ʻamakihi genome had been filtered to only include scaffolds ≥ 6,000 bp. 2425 of 13,551 scaffolds of our Oʻahu ʻamakihi reference genome were annotated.

Through the multispecies alignment process, Cactus reconstructs ancestral genome sequences for each node of the input guide tree. The split that gave rise to the ʻamakihi clade is hypothesized to have occurred ~2 million years ago, with Kauaʻi ʻamakihi diverging first and the split between Oʻahu ʻamakihi and Maui and Hawaiʻi ʻamakihi occuring ~1 million years ago (Lerner et al. 2011)—well before the introduction of avian malaria to the Hawaiian Islands. Therefore, to determine the ancestral allele for each SNP associated with malaria infection, we extracted the nucleotide at each SNP position from the ancestral reconstructed sequence for the Oʻahu, Maui, and Hawaiʻi ʻamakihi split, and from the ancestral reconstructed sequence for the split between Kauaʻi ʻamakihi and all other ʻamakihi species, nodes Anc39 and Anc37, respectively (Figure S3). We then used the base R function pairwise.prop.test (p.adjust.method = “BH”) to determine if genotypes homozygous for the ancestral or derived allele had lower infection prevalence at an adjusted *p‐value* ≤ 0.05.

We used BEDtools intersect v2.30.0 (Quinlan and Hall 2010) to determine if SNPs associated with infection fell within a gene or within 5 kb upstream or downstream of a transcription start site. If a SNP fell within a coding DNA sequence (CDS) interval that was in a multiple of three, we extracted the sequence and visually inspected the SNP position to determine if the polymorphism resulted in an amino acid change.

For SNPs that could not be annotated to either a gene or a transcription start site, flanking nucleotides 2 kb upstream and downstream of the SNP position were extracted from the Oʻahu ʻamakihi reference genome and used as input into nucleotide BLAST. BLASTn hits were filtered to only include sequences with an *e*‐value ≤ 1 × 10^−40^, a sequence identity > 95%, and alignment length > 1000 bp.

We determined if significant SNPs associated with infection were enriched or depleted for Biological Process Gene Ontology (GO) terms using R package LOLA v3.21 (Sheffield and Bock 2016) and a false discovery rate (FDR) of 5%. Rather than calculating GO term enrichment based on gene lists, as is done in tools like PANTHER (Mi et al. 2019), LOLA determines enrichment based on the number of overlaps between genomic regions of interest and GO term region databases. LOLA counts the number of overlaps and uses Fisher's exact test to assess significance. This approach avoids issues with reference list bias when calculating GO term enrichment using gene lists. Our LOLA database was constructed by extracting, per GO term, CDS and exon coordinates from the halLiftover Oʻahu ʻamakihi annotations. We used the database STRING to search for protein interactions between our annotated variants (Szklarczyk et al. 2023).

## 3 Results

### 3.1 Avian Malaria Prevalence in Oʻahu ʻamakihi

**FIGURE 2:**
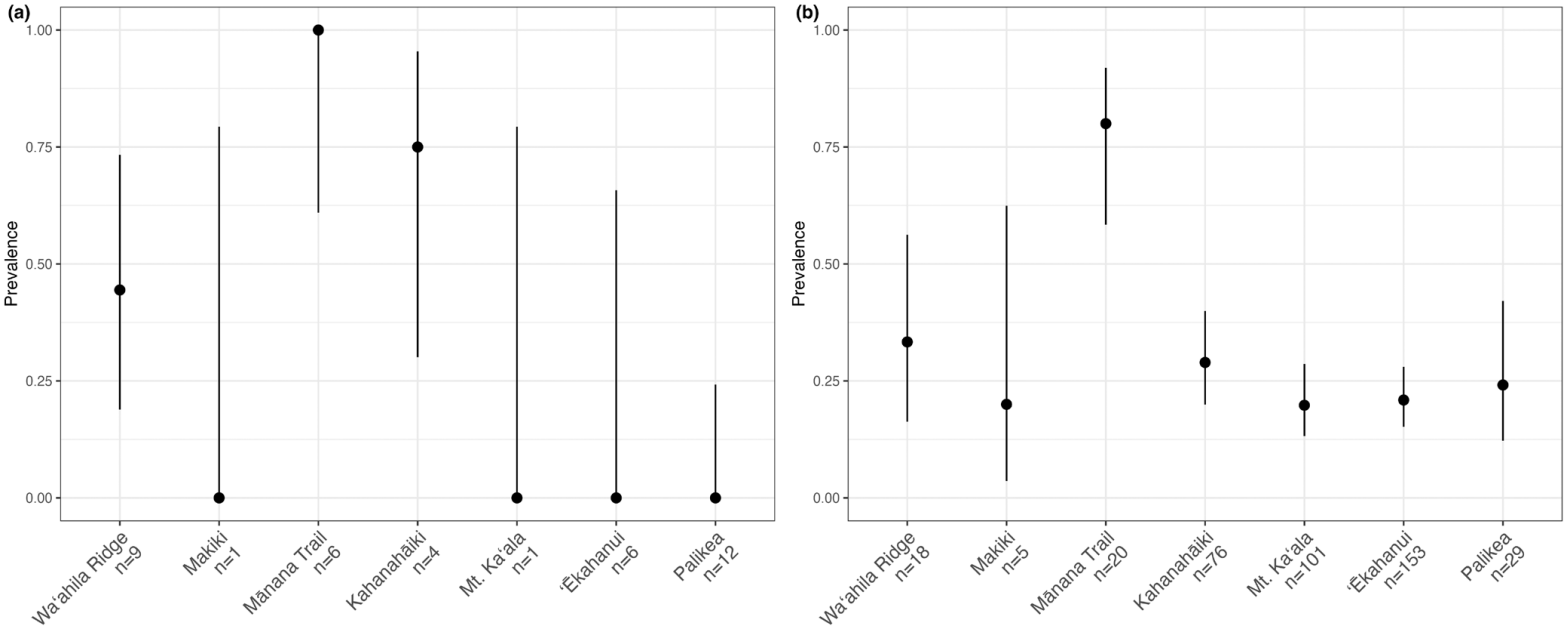
Sampling site Oʻahu ʻamakihi infection prevalence (a) and (b) malaria infection prevalence across all birds sampled. Points show avian malaria prevalence with 95% Wilson's confidence intervals. Figures (a) and (b) incorporate data from Neddermeyer et al. (2023).

We tested 36 Oʻahu ʻamakihi samples for avian malaria infection, 13 of which were positive. We found substantial variation in ʻamakihi prevalence across sites, ranging from no positive samples at Palikea to no negative samples at Mānana Trail (Figure 2a). However, apart from Mānana Trail, avian community malaria infection prevalence (malaria infection prevalence across all birds sampled) was consistent across sites (Figure 2b).

### 3.2 Sample Sequencing, SNP Detection and Genetic Diversity

We assembled a high‐quality *de novo* Oʻahu ʻamakihi genome, with scaffold and contig N50 of 12.99 MB and 117.2 KB, respectively, and a 95% BUSCO completeness score (Table S2). We retained 35 Oʻahu ʻamakihi samples across seven localities after removing those with mean sequencing depth < 4× and reference genome coverage < 85% (Figure 1; Table S1). Of these, 22 were malaria negative and 13 were positive. Mean genome‐wide sequence depth ranged from 4.82× to 63.52×, with reference genome coverage ranging from 87.9% to 96.7%. We were able to determine genotypes for 889,232,201 positions across our 1 GB reference genome for all samples, of which, after quality filtering, 5,770,073 were polymorphic. Genome‐wide heterozygosity across all samples ranged from 0.0019–0.003, with malaria‐negative sample mean heterozygosity 0.0023 (±1.6 × 10^−4^ SD) and malaria‐positive sample mean heterozygosity 0.0024 (±1.2 × 10^−4^ SD). There was no statistically significant difference in genome‐wide heterozygosity between malaria‐positive and malaria‐negative samples (Figure S4).

### 3.3 Oʻahu ʻamakihi Population Structure

We evaluated Oʻahu ʻamakihi population structure based on 426,241 linkage‐pruned SNPs. Though our cross‐validation results support two Oʻahu ʻamakihi populations (Figure S5), visual inspection of our ADMIXTURE analysis (Figure S6) and PCA are consistent with an isolation‐by‐distance pattern of a single Oʻahu ʻamakihi population (Figure 1). Furthermore, our PCA showed malaria‐positive and negative individuals clustered with their sampled populations rather than by infection status. Samples from Waʻahila Ridge and Palikea, the southern extent of the Koʻolau mountains on the eastern side and Waiʻanae mountains on the western side of Oʻahu, respectively, are genetically most distant from one another, and as populations move north within the Koʻolau and Waiʻanae mountain ranges, populations become more similar to populations on the opposite side of the island (Figure 1).

### 3.4 SNP Prevalence Association, Annotation, and GO Term Enrichment

**FIGURE 3:**
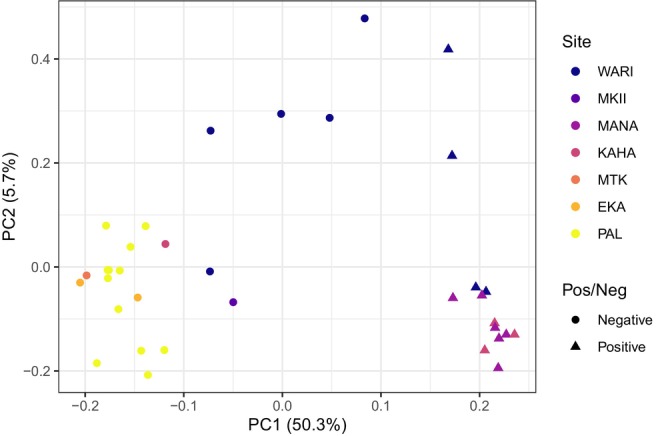
Exploratory principal component analysis (PCA) on 157 single nucleotide polymorphisms (SNPs) with F_ST_ > 0.35. F_ST_ threshold determined from 95% bias‐corrected and accelerated CI of genome‐wide F_ST_ values between infected and uninfected individuals. Site names and colors correspond to those on Figure 1. WARI = Waʻahila Ridge, MKII = Makiki, MANA = Mānana Trail, KAHA = Kahanahāiki, MTK = Mt. Kaʻala, EKA = ʻĒkahanui, PAL = Palikea. Circles and triangles symbolize whether a sample was PCR negative or positive for avian malaria.

**FIGURE 4:**
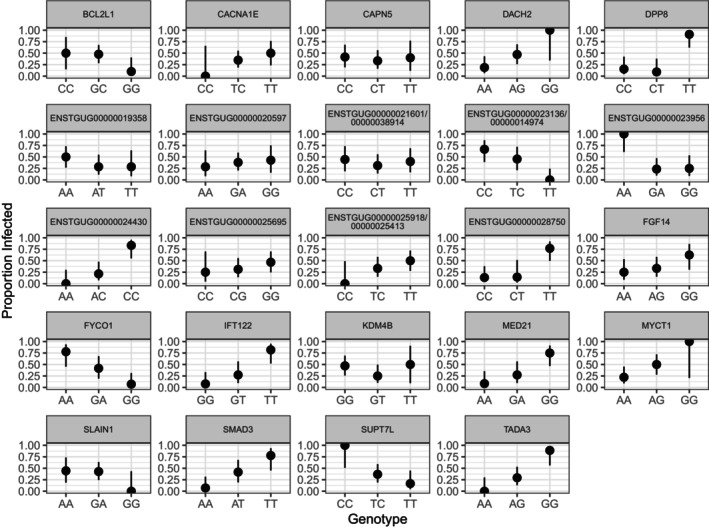
Annotated avian malaria infection‐associated genotypes found within genes based on the top 0.1% of significant variants from the linear mixed model, significant variants from the linear model, and pairwise F_ST_ comparisons between infected and uninfected individuals. Points show infection prevalence with 95% Wilson's confidence intervals. Panels represent the gene or locus associated with variants. Note variants annotated to TADA3 and ENSTGUG00000028750 were also annotated to a transcription start site for a locus described in Table 2.

**FIGURE 5:**
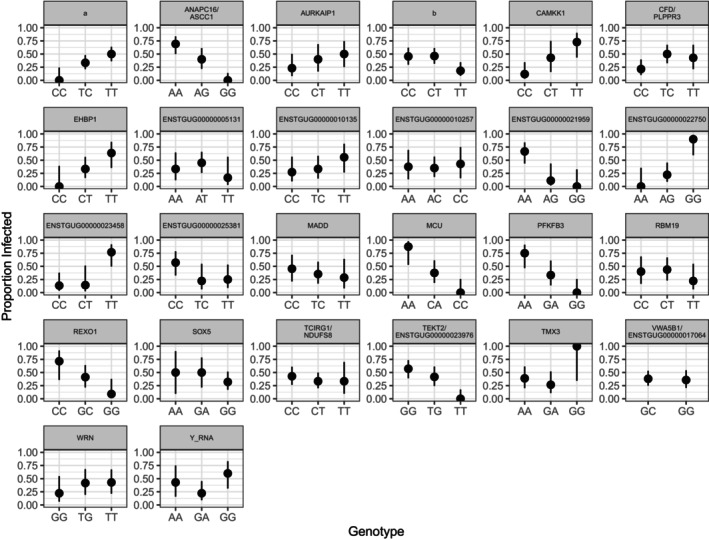
Transcription start site (TSS) annotated avian malaria infection‐associated genotypes based on the top 0.1% of significant variants from the linear mixed model and pairwise F_ST_ comparisons between infected and uninfected individuals. Points show infection prevalence with 95% Wilson's confidence intervals. Panels represent gene or locus names associated with TSS variants. Variants were annotated to a TSS if the variant fell within 5 kb upstream or downstream of a TSS. Panel (a) represents gene IDs ENSTGUG00000025918, ENSTGUG00000007721, and ENSTGUG00000025413, and panel (b) represents gene IDs ENSTGUG00000027186, ENSTGUG00000029360, and ENSTGUG00000027571.

Using our linkage‐pruned SNP set, we found per‐SNP F_ST_ values between infected and uninfected individuals ranged from 0 to 0.49, with a median of 0, a mean of 0.018, and 95% CI upper limit of 0.35. For illustrative purposes, we extracted 157 positions with F_ST_ values that exceeded the upper 95% CI and ran a PCA on those sites. The isolation‐by‐distance pattern observed in Figure 1 was no longer evident, and samples clustered based on infection status along PC 1, which explained 50.3% of variation (Figure 3). We were able to annotate 15 of 157 SNPs exceeding the 95% CI; five were in 3′ and 5′ untranslated regions (UTR), two were in exons of long non‐coding RNAs (ncRNAs), two were in CDS, and eight were in putative regulatory regions (Figures 4 and 5; Tables 1 and 2). Variant simple_8802:56527 within a 3′ UTR was annotated to two different transcripts that coded for different genes. Several variants within putatively regulatory regions (simple_7:1059325, simple_7:8415740, and simple_351:34779) were associated with multiple genes, and two of these (simple_7:1059325 and simple_7:8415740) were also annotated within genes (Table 2). No variant exhibited heterozygote advantage, where heterozygous individuals exhibited reduced malaria prevalence compared to homozygous individuals (Figures 4 and 5). Heterozygous individuals at 12 of 15 positions exhibited intermediate phenotypes, where homozygous individuals had either higher or lower infection prevalence, and for three variants, heterozygous individuals had similar prevalence to one of two homozygous individuals (Figures 4 and 5).

**TABLE 1:** Single nucleotide polymorphisms (SNPs) annotated within genes associated with Oʻahu ʻamakihi infection prevalence.

Significance test	SNP	Ancestral allele	Gene ID	Sequence type	Gene name	Beta	Stdv	Description
Linear Mixed Model Likelihood Ratio Test	simple_10:615342	T	ENSTGUG00000017352	CDS	CACNA1E	−0.484	0.14	Governs calcium ion transport into the cell
	simple_200:4039	T	ENSTGUG00000025918/ENSTGUG00000025413	Exon	—	−0.186	0.13	Long non‐coding RNA
	simple_257:1544928	C	ENSTGUG00000013052	3′ UTR	CAPN5	−0.405	0.11	Belongs to a group of genes called calpains that are calcium‐dependent cysteine proteases
	simple_349:168327	C	ENSTGUG00000006677	3′ UTR	BCL2L1	0.464	0.12	Plays a major role in the inhibition of apoptosis
	simple_40:14399153	G^a^	ENSTGUG00000023956	Exon	—	0.323	0.11	Long non‐coding RNA
	simple_400:1059598	C	ENSTGUG00000025695	Exon	—	0.324	0.12	Involved in nucleotide synthesis
	simple_41:25326245	G	ENSTGUG00000020597	Exon	—	0.190	0.11	Long non‐coding RNA
	simple_51:46446977	G	ENSTGUG00000012572	CDS	SLAIN1	−0.353	0.10	Involved in microtubule organization
	simple_67:42015240	A	ENSTGUG00000019358	Exon	—	−0.271	0.10	Long non‐coding RNA
	simple_6731:17343	T^a^	ENSTGUG00000002523	3′ UTR	SUPT7L	0.395	0.11	Involved in the regulation of DNA transcription through chromatin modification
	simple_85:493992	G	ENSTGUG00000000775	3′ UTR	KDM4B	−0.163	0.13	Involved in the regulation of DNA transcription through chromatin modification
Linear Model Likelihood Ratio Test	simple_334:1599110^c^	T	ENSTGUG00000021601/ENSTGUT 00000038914	Exon	—	0.441	0.08	Novel Ensembl gene with no known current function
	simple_51: 35886439^c^	G	ENSTGUG00000010862/ENSTGUT00000011318	Exon	FGF14	0.434	0.08	Involved in a wide array of cell functions, including cell division and survival, cell growth, and tissue repair. Implicated in cerebral malaria neuroinflammation
	simple_59:3744831^d^	A	ENSTGUG00000002423/ENSTGUT00000002525	Exon	DACH2	0.581	0.10	Involved in transcription regulation and regulation of organogenesis and myogenesis
	simple_67:6286879^c^	G	ENSTGUG00000011193/ENSTGUT00000011660	Exon	MYCT1	0.588	0.11	Regulates hematopoietic stem cell homeostasis, apoptosis, and endocytosis
F_ST_ ≥ 0.35 (Upper 95% CI)	simple_121:279458	G^a^	ENSTGUG00000010483	CDS	IFT122	—	—	Plays a role in primary cilium assembly and protein transport to the cilium
	simple_13:5436421	G^a^	ENSTGUG00000004520	5′ UTR	FYCO1	—	—	Involved in autophagosome transport along microtubules
	simple_20:15648445	A^a^	ENSTGUG00000024373	3′ UTR	MED21	—	—	Involved in the regulation of RNA polymerase II
	simple_51:1840649	C^b^	ENSTGUG00000024430	Exon	—	—	—	Long non‐coding RNA
	simple_52:15999285	C^a^	ENSTGUG00000009145	3′ UTR	DPP8	—	—	Involved in the regulation of programmed cell death
	simple_52:16890071	A^a^	ENSTGUG00000009470	3′ UTR	SMAD3	—	—	Involved in a wide array of cell functions, regulating gene activity by transmitting cell surface signals to the nucleus. Through transforming growth factor‐β, SMAD3 has been shown to regulate T‐cell activation
	simple_7:1059325	A^a^	ENSTGUG00000008689	CDS	TADA3	—	—	An adaptor protein involved in a wide array of processes associated with gene expression. Including the regulation of the p53 tumor suppressor protein
	simple_7:8415740	T^b^	ENSTGUG00000028750	Exon	—	—	—	Long non‐coding RNA
	simple_8802:56527	T^a^	ENSTGUG00000023136/ENSTGUG00000014974	3′ UTR	—	—	—	Involved in proteolysis

^a^ Indicates homozygous ancestral allele infection prevalence is significantly lower than homozygous derived allele infection prevalence based on a pair‐wise proportion test false discovery rate adjusted *p*‐value ≤ 0.05.

^b^ Indicates homozygous derived allele infection prevalence is significantly lower than homozygous ancestral allele infection prevalence based on a pair‐wise proportion test false discovery rate adjusted *p*‐value ≤ 0.05.

^c^ *q*‐value < 0.30.

^d^ *q*‐value < 0.10.

**TABLE 2:** Transcription start site (TSS) annotated single nucleotide polymorphisms (SNPs) associated with Oʻahu ʻamakihi infection prevalence. Variants were annotated to a TSS if the variant fell within 5 kb upstream or downstream of a TSS.

Significance test	SNP	Ancestral allele	Gene ID	Gene name	Beta	Stdv	Description
Linear Mixed Model Likelihood Ratio Test	simple_102:232823	C	ENSTGUG00000010135	—	0.162	0.09	Novel ensembl gene related to microtubule cytoskeleton organization
	simple_123:303619	C	ENSTGUG00000007403/ENSTGUG00000007436	TCIRG1/NDUFS8	0.443	0.11	TCIRG1 plays a role in intracellular pH regulation on the cell surface. NDUFS8 is a mitochondrial membrane protein involved in electron transport
	simple_14:1737878	T	ENSTGUG00000001765	WRN	0.150	0.09	WRN plays a role in DNA repair and maintenance
	simple_15:5343099	C	ENSTGUG00000009017	RBM19	0.163	0.09	RBM19 codes for RNA‐binding motifs and may be involved in regulating ribosome biogenesis
	simple_15:7561586	A	ENSTGUG00000010257	—	0.262	0.13	Cell membrane protein facilitating glucose transport
	simple_16:820304	G^b^	ENSTGUG00000023976/ENSTGUG00000001625	TEKT2	−0.209	0.10	TEKT2 belongs to a family of filament‐forming proteins associated with the formation of ciliary and microtubules
	simple_18:5961010	A	ENSTGUG00000017662	Y_RNA	−0.287	0.11	Small non‐coding RNA involved in DNA replication
	simple_200:4039	T^b^	ENSTGUG00000025918/ENSTGUG00000007721/ENSTGUG00000025413	—	−0.186	0.13	Long non‐coding RNA
	simple_20:835613	G	ENSTGUG00000011749	SOX5	0.423	0.11	Involved in transcription regulation
	simple_23:1022016	T	ENSTGUG00000002228	EHBP1	−0.380	0.11	Involved in endocytic trafficking and actin reorganization
	simple_25:131767	A	ENSTGUG00000009298	TMX3	0.381	0.12	Endoplasmic reticulum membrane protein associated with reducing the impacts of oxidative stress
	simple_270:926105	G	ENSTGUG00000017103/ENSTGUG00000017064	VWA5B1	−0.398	0.15	ENSTGUG00000017064 is associated with mRNA export from the nucleus. In humans, VWA5B1 plays a role in blood clotting
	simple_276:464227	C	ENSTGUG00000000652/ENSTGUG00000000656	PLPPR3/CFD	0.318	0.08	PLPPR3 is in the lipid phosphate phosphatase protein family of membrane proteins involved in modulating lipid phosphates. CFD encodes for complement factor D, a crucial protein in the complement system
	simple_294:64159	C	ENSTGUG00000027186/ENSTGUG00000029360/ENSTGUG00000027571	—	−0.316	0.09	ENSTGUG00000027186 and ENSTGUG00000029360 are novel Ensembl genes with no known current function. ENSTGUG00000027571 is a long non‐coding RNA
	simple_32:5762615	A	ENSTGUG00000005131	—	0.284	0.14	Involved in protein post‐translational modifications
	simple_357:172788	T	ENSTGUG00000003131	AURKAIP1	0.329	0.09	Involved in the regulation of proteolysis
	simple_42:2390082	T	ENSTGUG00000010454	MADD	−0.347	0.09	Involved in the regulation of apoptosis
	simple_47:682337	G	ENSTGUG00000000250	REXO1	0.337	0.09	Involved in nucleic acid degradation
	simple_67:1081934	T	ENSTGUG00000025381	—	−0.253	0.09	p53 apoptosis effector related to PMP‐22‐like
F_ST_ ≥ 0.35 (Upper 95% CI)	simple_32:3988866	C^a^	ENSTGUG00000004191	CAMKK1	—	—	Associated with a class of enzymes regulated by intracellular calcium concentrations. Playing a role in breakdown of ATP
	simple_346:687280	A^b^	ENSTGUG00000021959	—	—	—	Long non‐coding RNA
	simple_351:219840	C^a^	ENSTGUG00000004428	MCU	—	—	Related to mitochondrial calcium ion regulation
	simple_351:34779	A^b^	ENSTGUG00000004483/ENSTGUG00000022475	ASCC1/ANAPC16	—	—	ASCC1 is a part of a transcriptional coactivator that plays a role in gene transactivation. ANAPC16 is a promoter involved in cell division
	simple_57:22397165	G^a^	ENSTGUG00000002025	PFKFB3	—	—	Regulates cellular glucose metabolism
	simple_7:1059325^c^	A^a^	ENSTGUG00000006159/ENSTGUG00000008730/ENSTGUG00000025146/ENSTGUG00000026741/ENSTGUG00000008649	CAMK1	—	—	ENSTGUG00000006159 is associated with microtubule glycylation of primary cilia, ENSTGUG00000008730 is a pseudogene, ENSTGUG00000025146 and ENSTGUG00000026741 are associated with cilium assembly and movement, and CAMK1 is associated with an intracellular calcium signaling cascade involved in phosphorylation
	simple_7:3442307	G^b^	ENSTGUG00000022750	—	—	—	Long non‐coding RNA
	simple_7:8415740^c^	T^b^	ENSTGUG00000023458	—	—	—	Long non‐coding RNA

^a^ Indicates homozygous ancestral allele infection prevalence is significantly lower than homozygous derived allele infection prevalence based on a pair‐wise proportion test false discovery rate adjusted *p*‐value ≤ 0.05.

^b^ Indicates homozygous derived allele infection prevalence is significantly lower than homozygous ancestral allele infection prevalence based on a pair‐wise proportion test false discovery rate adjusted *p*‐value ≤ 0.05.

^c^ Variant was also found within a locus described in Table 1.

Our LMM, based on 426,241 linkage‐pruned and filtered SNPs, explained 50% (± 20.1% SE) of phenotypic variation, of which 12% was explained by SNPs with nonzero effects, with population structure potentially explaining the remaining 35%. At a Bonferroni‐corrected *p*‐value ≤ 1.17 × 10^−7^, we identified 36,541 SNPs significantly associated with infection. Due to limited sample size and differences in Oʻahu ʻamakihi infection prevalence between our two most genetically distinct populations, Palikea and Waʻahila Ridge (Figures 1 and 2), our analysis likely identified many false positives. To reduce false positives associated with population structure, we focused our analysis on 425 variants, the top 0.1% positions significantly associated with infection (Figure S7). We were able to annotate 30 of the 425 SNPs: four were in 3′ UTRs, two were in CDS, five were in exons, of which four were long ncRNAs, and 19 were in putative regulatory regions (Figures 4 and 5; Tables 1 and 2). Variant simple_200:4039 was found in an exon of two different long ncRNA molecules. Several LMM variants within putatively regulatory regions (simple_123:303619, simple_16:820304, simple_200:4039, simple_270:926105, simple_276:464227, and simple_294:64159) were associated with multiple genes (Table 2). Similar to our F_ST_ results, heterozygous individuals largely exhibited an intermediate phenotype where homozygous individuals had either higher or lower infection prevalence, or heterozygous individuals had similar prevalence to one of two homozygous genotypes (Figures 4 and 5). Heterozygous individuals for variants associated with two genes, Lysine Demethylase 4B (KDM4B), Thioredoxin Related Transmembrane Protein 3 (TMX3), and a Y RNA had lower infection prevalence (Figures 4 and 5). Of all annotated SNPs, only one position, simple_270:926105, was missing one of two homozygous genotypes, CC, entirely across all 35 samples. Simple_270:926105 is in a putative regulatory region for Von Willebrand Factor A Domain Containing 5B1 (VWA5B1), which plays a role in blood clotting in humans. In malaria mouse models, von Willebrand factor‐negative mice show shortened survival and higher parasite load (Kraisin et al. 2019). The LM identified four SNPs at *q‐*value ≤ 0.30 and three with *q‐*value ≤ 0.10 (Figure S8). We annotated four of the seven SNPs, all of which fell within exons (Table 1), and all heterozygous individuals exhibited an intermediate phenotype, whereas homozygous individuals had either higher or lower infection prevalence (Figure 4). All seven SNPs were significant in the LMM at a Bonferroni‐corrected *p*‐value ≤ 1.17 × 10^−7^. Our BLASTn query annotated an additional nine out of the 589 significant SNPs across our LMM, LM, and F_ST_ analyses (Table S3), bringing the total number of annotated SNPs to 58. The genomic inflation factor for our LMM, 3.62, and Q–Q plot (Figure S9) indicates there is *p‐*value inflation due to the inability of the relatedness matrix to account for Oʻahu ʻamakihi population structure. In our LMM, we were unable to control for population structure by adding PCs. Even with the inclusion of just principal component 1, our model quickly became oversaturated, likely due to small sample size. Conversely, the genomic inflation factor for our LM, 1.06, and Q–Q plot (Figure S10) did not indicate model violations due to hidden population structure.

**FIGURE 6:**
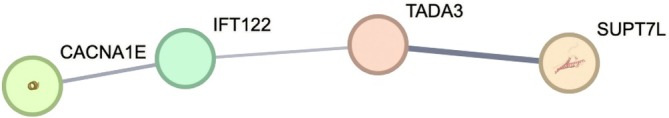
Output of exploratory protein–protein interaction network analysis of annotated variants associated with avian malaria infection. Protein–protein interaction *p*‐value 0.026 between SUPT7L, TADA3, IFT122, and CACNA1E. Line thickness represents the strength of data supporting the interaction.

**FIGURE 7:**
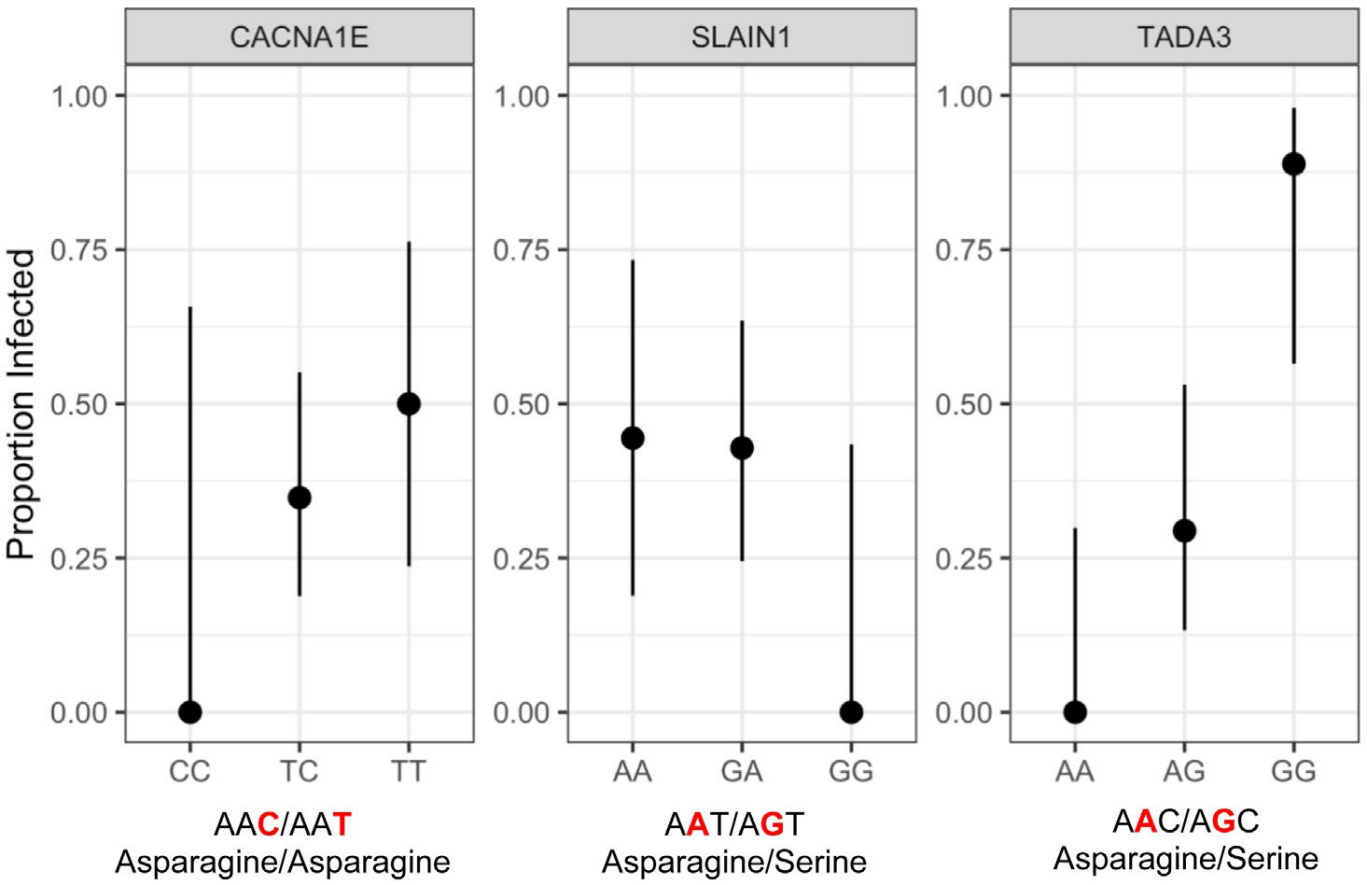
Genotype by avian malaria infection prevalence for variants associated with infection based on F_ST_ and linear mixed model analyses that were located within coding DNA sequences. Labels beneath the x‐axis indicate codon and corresponding amino acid for each variant. Bold red letters indicate SNP codon position. Points show infection prevalence with 95% Wilson's confidence intervals. Panels represent the gene name to which the variant was annotated.

Across 58 significant SNPs with annotations, STRING suggested potential protein interaction enrichment between SPT7 Like, STAGA Complex Subunit Gamma (SUPT7L), Transcriptional Adaptor 3 (TADA3), Intraflagellar Transport 122 (IFT122), and Calcium Voltage‐Gated Channel Subunit Alpha1 E (CACNA1E) (protein–protein interaction, *p* = 0.026; Figure 6). We were able to evaluate whether a variant resulted in an amino acid change for three of four SNPs found within CDS, SLAIN Motif Family Member 1 (SLAIN1), TADA3, and CACNA1E. Two polymorphisms were located at 2nd codon positions, one within SLAIN1 and one within TADA3, resulting in an amino acid change from asparagine to serine, and one polymorphism within CACNA1E was located at the 3rd codon position, resulting in no amino acid change (Figure 7). At a 10% FDR, our LOLA analysis did not find any significantly enriched or depleted gene ontology terms.

Our proportion tests of annotated SNPs, found 19 positions where there was a significant difference between the proportion of infected individuals and homozygous genotype. At nine positions within genes, individuals homozygous for the ancestral allele had lower infection prevalence, and at two positions within genes, individuals homozygous for the derived allele had lower infection prevalence (Table 1; Table S4). When looking at SNPs within putative *cis*‐regulatory regions, we found the inverse pattern, with three positions where individuals homozygous for the ancestral allele had lower infection prevalence and five positions where individuals homozygous for the derived allele had lower infection prevalence (Table 2; Table S4). For the seven positions with low‐prevalence homozygous derived alleles, six were associated with long ncRNAs, whereas only one of the 12 positions with low‐prevalence homozygous ancestral alleles was associated with long ncRNAs (Tables 1 and 2). Further, along the suggestive STRING protein–protein interaction network, three of four genes, SUPT7L, TADA3, and IFT122, had lower infection prevalence in individuals homozygous for the ancestral allele (Figure 6 and Table 1). Across all annotated SNPs, five associated with infection, ENSTGUG00000019358, WRN, IFT122, KDM4B, and VWA5B1, had a different reconstructed ancestral allele between Anc39 and Anc37, with only position simple_121:279458, gene IFT122, having a significant difference in infection prevalence between homozygous genotypes (Table S5). Given the overall consistency between reconstructed ancestral alleles for Anc39 and Anc37, we chose to use Anc39 in our analysis, given that the reconstruction represents the Oʻahu ʻamakihi's most recent common ancestor (Figure S3).

## 4 Discussion

We used whole‐genome resequencing data of 35 Oʻahu ʻamakihi to identify 589 SNPs associated with avian malaria infection. Annotations for 58 of those 589 variants revealed genes associated with transcription regulation, DNA repair, reducing oxidative stress, cellular glucose regulation, von Willebrand factor protein, complement, apoptosis, T‐cell activation, autophagosome assembly and transport, and cellular calcium transport, as well as long non‐coding and Y RNA molecules (Tables 1 and 2). Further, we found that Oʻahu ʻamakihi population structure is consistent with an isolation‐by‐distance pattern between the Koʻolau and Waiʻanae mountains (Figure 1), but visual inspection of a SNP infection‐associated PCA shows the isolation pattern broke down and samples from different mountain ranges clustered based on infection status (Figure 3).

*Plasmodium* species are intracellular parasites, and to replicate successfully will co‐opt host cellular machinery. Regulation of host cell calcium ion concentrations is important for stimulating multiple phases of parasite replication, from asexual reproduction to egress (Brochet and Billker 2016; Cheemadan et al. 2014), and as a result, *Plasmodium* has been shown to increase cell calcium ion concentrations (Alleva and Kirk 2001; Leida et al. 1981; Tanabe et al. 1982). We identified five variants associated with genes involved in intracellular calcium responses; two were found within genes, CACNA1E and calpain‐5 (CAPN5), and three were found in gene‐associated *cis*‐regulatory regions, calcium/calmodulin‐dependent protein kinase kinase 1 (CAMKK1), calcium/calmodulin‐dependent protein kinase 1 (CAMK1), and mitochondrial calcium uniporter (MCU). These genes play roles in intracellular calcium regulation, from mediating the entry of calcium into cells and mitochondria to triggering apoptosis in the presence of excess calcium (Momeni 2011; Sayers et al. 2022). A suggested protein interaction network analysis identified a potential axis along which calcium ion regulation may be acting between SUPT7L, TADA3, IFT122, and CACNA1E (Figure 6). SUPT7L and TADA3 are associated with transcription regulation (Sayers et al. 2022), and IFT122 belongs to a complex of genes involved in mediating ciliary G protein‐coupled receptor trafficking (Hirano et al. 2017), including trafficking of intracellular calcium (Dhyani et al. 2020; Mykytyn and Askwith 2017). The protein–protein interaction network results are promising; further studies with larger sample sizes are needed to confirm these conclusions. Genotypes associated with low prevalence may be better equipped to control parasite replication via regulation of calcium ion influx into infected cells. We found two variants along the protein interaction network within CDS, one in TADA3 and one in CACNA1E. The SNP within TADA3 resulted in two different amino acids, asparagine and serine, whereas the SNP found in CACNA1E did not result in an amino acid change (Figure 7). Synonymous variants have been shown to impact transcription efficiency and accuracy, with different codons improving or hindering amino acid translation (Hunt et al. 2014; Moura et al. 2011; Walsh et al. 2020).

Autophagy is a major lysosome degradative pathway by which the cell disposes of damaged organelles and plays a role in adaptive and innate immune signaling (Beau et al. 2011). Microtubules play an important role in the autophagy process through the assembly and transport of autophagosomes to lysosomes (Mackeh et al. 2013; Monastyrska et al. 2009), making microtubules a target of *Plasmodium* antagonism (Vijayan et al. 2022). After cell invasion, *Plasmodium* replicates inside a parasitophorous vacuole membrane (PVM). PVMs are the target of host autophagy markers in human models of *Plasmodium* infection (Agop‐Nersesian et al. 2018; Thieleke‐Matos et al. 2016). In response, *Plasmodium* has been shown to produce an inhibitory protein to avoid PVM removal via autophagy (Real et al. 2018). We identified five variants associated with genes that could play a role in the transport of autophagosomes via microtubules in response to malaria infection. Two variants were found within genes, SLAIN1 and FYVE and Coiled‐Coil Domain Autophagy Adaptor 1 (FYCO1), and three in gene‐associated cis‐regulatory regions, ENSTGUG00000010135, Tektin 2 (TEKT2), and ENSTGUG00000006159. The SLAIN1 variant associated with infection was nonsynonymous, resulting in two amino acid changes. FYCO1 is implicated in microtubule transport of autophagosomes (Sayers et al. 2022). *Plasmodium* may indirectly inhibit autophagosome transport via FYCO1 by interacting with marker LC3 present on autophagic vesicles (Sayers et al. 2022).

Apoptosis is a form of programmed cell death and has long been known to play a role in managing malaria infection (Sena‐Dos‐Santos et al. 2021). We evaluated four variants associated with apoptosis, two within genes that contribute to apoptosis regulation (BCL2L1 and DPP8) and two in gene‐associated *cis*‐regulatory regions (MADD and ENSTGUG00000025381). One of which, DPP8, has been implicated in the regulation of inflammasomes (Johnson et al. 2020; Okondo et al. 2018). In response to infection, inflammasomes can trigger a specific type of apoptotic process, pyroptosis (de Zoete et al. 2014; Kuriakose and Kanneganti 2023; Sena‐dos‐Santos et al. 2023; Yu et al. 2021). In human *Plasmodium vivax* infections, mutations in inflammasome‐related genes were related to infection outcomes (Santos et al. 2016), suggesting this mechanism could be evolutionarily conserved. In addition, B‐cell Lymphoma 2 genes are important for blocking apoptotic signaling in the mitochondria (Reed et al. 1998). In human malaria models, *Plasmodium* inhibits apoptosis in infected cells (Leirião et al. 2005; van de Sand et al. 2005). BCL2 function was found to be inhibited by *Plasmodium*, and when function was restored, apoptosis occurred preferentially in malaria‐infected cells (Kaushansky et al. 2013). However, when the effect of host BCL2 genotype on human malaria parasite density was tested, no effect was found (Sena‐dos‐Santos et al. 2023).

We identified six variants within exons of long ncRNA, and five variants in long ncRNA‐associated *cis*‐regulatory regions correlated with avian malaria infection. Gene expression can be modified by non‐coding RNA molecules (Mercer et al. 2009). During infection, malaria impacts host regulation of long ncRNAs (Chen et al. 2022; Oboh et al. 2024). The transforming growth factor‐β (TGF‐β)/SMAD3 immune signaling pathway is potentially regulated by host long ncRNA molecules during *Plasmodium* infection (Chen et al. 2022). TGF‐β/Smad3 signaling is involved in T‐cell activation (Delisle et al. 2013) and antimalarial immunity (Chen et al. 2022). Here, a variant within SMAD3 was associated with Oʻahu ʻamakihi avian malaria infection. One potential hypothesis is that shifts in the regulation of SMAD3 signaling by long ncRNA molecules in infected ʻamakihi may act to limit parasite replication.

Within a putatively *cis*‐regulatory region, we identified one variant associated with the immune response that directly interacts with pathogen surface molecules, Complement Factor D (CFD). The complement system is involved in both the adaptive and innate immune response (Dunkelberger and Song 2010). One annotated variant, simple_270:926105, was missing one of two homozygous genotypes across all 35 samples. The position was found within a *cis*‐regulatory region associated with von Willebrand Factor A Domain Containing 5B1 (VWA5B1). Von Willebrand Factors play an important role in the clotting cascade (Sadler 1998), and in mouse models of *Plasmodium* infection, variants in von Willebrand Factor genes are associated with higher parasitemia and anemia (Kraisin et al. 2019). The complete absence of a CC genotype in our 35 samples may indicate that the allele in homozygotes is strongly deleterious in Oʻahu ʻamakihi. Greater sampling of Oʻahu ʻamakihi populations can determine if this genotype is completely absent or exists at low frequencies across the island.

Modifications to *cis*‐regulatory regions play an important role in biological evolution (He et al. 2021; Krishnan et al. 2022; Roscito et al. 2018). We found annotations related to intracellular calcium ion regulation and response, primary cilia movement and organization, microtubule organization and transport, and long ncRNA molecules overlapping between variants found both within genes and within putative cis‐regulatory regions. Identifying genes involved in these pathways in both a regulatory and within‐gene context lends support to the importance of these pathways in managing avian malaria infection. Three variants within modeled *cis*‐regulatory regions were annotated to more than two gene IDs (Tables 1 and 2). Two variants, simple_7:8415740 and simple_7:1059325, were found to be both within genes and *cis*‐regulatory regions; however, both intragenic and *cis*‐regulatory annotations had different gene IDs (Tables 1 and 2). This may be due to a combination of annotation errors and hotspots in regulatory regions where multiple genes share cis‐regulatory elements.

We conducted exploratory analyses on 19 positions where individuals with one of two homozygous genotypes had lower infection prevalence (Tables 1 and 2). Within genes, genotypes with low infection prevalence tend to be homozygous for the ancestral allele (9 of 11 significant sites), whereas within putative *cis*‐regulatory regions, genotypes with low infection prevalence tend to be homozygous for the derived allele (5 of 8 significant sites). Further, low‐prevalence homozygous derived allele genotypes were often associated with long ncRNAs (6 of 7 positions). Genomic regions that play a role in gene regulation may be more tolerant of novel mutations than intragenic regions, offering the opportunity for the evolution of beneficial genotypes. Here, we identify potential evolution of beneficial genotypes within *cis*‐regulatory regions and long ncRNA molecules in the context of avian malaria infection in Oʻahu ʻamakihi. We also find evidence of potential relaxation of selection due to the historical absence of avian malaria. Reduced selective pressures may have allowed the accumulation of deleterious mutations in intragenic regions important in combating infection. Often, individuals homozygous for the derived allele within intragenic regions had higher infection prevalence.

The results from three studies focused on Hawaiʻi ʻamakihi using target‐capture‐seq (Cassin‐Sackett et al. 2019) and infection‐associated transcriptome sequencing (Cassin‐Sackett et al. 2025; K. L. Paxton et al. 2023) identified intracellular calcium signaling as likely being under selection from exposure to avian malaria. Two of which also identified von Willebrand Factor genes as being important in the infection response (Cassin‐Sackett et al. 2025; K. L. Paxton et al. 2023). Although there was no overlap in specific genes being linked to malaria resistance between our study and this previous work, we did find intracellular calcium signaling and von Willebrand Factors to be significantly associated with malaria infection in Oʻahu ʻamakihi. The response to selective pressure that malaria exerts on hosts is likely polygenic. Even within Hawaiʻi ʻamakihi, different populations appear to have different ways of managing malaria infection (Cassin‐Sackett et al. 2019). Therefore, it is not unlikely that different genes, but similar pathways across closely related species, would be under selection to manage malaria infection. Further, our limited sample size, 35 samples with 13 infected individuals, presents two statistical problems: false positives due to effect size inflation and reduced statistical power. Through random chance, SNPs in GWAS can have artificially inflated effect sizes and therefore have lower *p*‐values. SNPs with the most artificially inflated effect sizes might be identified as the most significantly associated with a phenotype (Winner's Curse), making reproducibility a challenge (Sidebotham and Barlow 2024). As sample size increases, the likelihood of this phenomenon decreases. Given our sample size constraints and corresponding reduced statistical power, our analysis may have missed SNPs that are associated with infection but were not identified. Contrary to expectations, our LMM likely had far more false positive SNPs than our LM (Figures S9 and S10). This is likely the result of inflated effect sizes due to SNPs clustering based on population structure. The most genetically distinct ʻamakihi populations had some of the largest differences in infection prevalence. Although there were ʻamakihi populations that had no infected individuals, avian malaria was present at every sampled site in almost equal proportions (Figure 2). More sampling is required to further separate effects of population structure from avian malaria.

Given the ubiquity of avian malaria across all sample sites (Figure 2), previous work on Oʻahu finding that, regardless of age class (e.g., juvenile vs. adult), individuals were equally likely to be infected (Neddermeyer et al. 2023), and the overall high Oʻahu ʻamakihi infection prevalence (36%; 95% CI 22.5%–52.4%), it is likely that PCR‐negative individuals have been exposed to avian malaria. However, given the complexity of the parasite life cycle, we cannot say that parasite clearance has been achieved. Evidence suggests that Hawaiʻi ʻamakihi maintain chronic infections (C. T. Atkinson et al. 2001), and parasite sequestration in non‐erythrocyte tissues may cause false‐negative PCR results from blood samples. Though anemia, caused by the destruction of red blood cells, is one of the more serious signs of disease in *Plasmodium*‐infected birds, and often results in death (C. T. Atkinson et al. 1995, 2000; Yorinks and Atkinson 2000). Through the reduction of blood parasite loads below our limit of detection, individuals fending off infection are likely preventing the most serious disease outcomes. Using cycle threshold values from our real‐time PCR, we visualized the relationship between *Plasmodium* DNA concentration and genotype (Figures S11 and S12). Although no immediate patterns were present, evaluating infection intensity by genotype may be an avenue for future research.

Correlational studies like ours cannot determine mechanisms of resistance, but these results lay the groundwork for targeted hypothesis testing and functional validation. This includes evaluating how calcium flux into infected cells and the regulation of host genes via long ncRNA molecules impact parasite replication. We sought to identify associations between genotype and avian malaria infection in 35 sampled individuals. Given the limited sample size, these analyses serve as a first step toward identifying plausible mechanisms of infection control that could be validated with follow‐up studies and more robust sample sizes. Further, visualization of our F_ST_ analysis showed clear clustering of individuals by infection status (Figure 3). However, F_ST_ comparisons can be highly influenced by uneven sampling and population structure independent of phenotypes of interest. To date, much of the literature on mechanistic responses to malaria infection and studies of avian genotype to phenotype associations more broadly rely on assumptions of gene function homology between mammals and birds. Although this is a solid starting point, it highlights the importance of functional validation for the associations presented here.

Avian malaria infection prevalence can vary substantially between years on Oʻahu (Neddermeyer et al. 2023). This inter‐year variability allows for the opportunity to evaluate whether allele frequencies at these loci will change between high and low infection years. For example, if there are fitness costs to having a malaria‐resistant genotype when infection is reduced across the landscape, there may be a change in allele frequency at infection‐prevalence‐correlated loci. If the importance of these loci are validated further, genotypes at infection‐associated loci may be worth considering when selecting individuals for Hawaiian honeycreeper captive breeding programs. Position simple_270:926105, in particular, may prove fruitful if more extensive sampling supports the absence of genotype CC; this may indicate the presence of a lethal allele at this position.

Variants were primarily associated with regulating parasite growth by acting on intracellular processes. Host limitations on *Plasmodium* replication, therefore, may not solely depend on the immune response but can also involve restricting intracellular components essential for completion of the *Plasmodium* life‐cycle. More generally, adaptation to introduced pathogens may primarily occur via reducing pathogen growth through non‐immune mechanisms. Immune‐mediated pathology is one of the main causes of disease during infection (Cao et al. 2023; Damjanovic et al. 2012; Mortaz et al. 2012) and is particularly important in determining human malaria infection outcomes (Artavanis‐Tsakonas et al. 2003; Coban et al. 2018). Thus, in response to novel infectious diseases, hosts may be more likely to evolve infection survival pathways that do not rely on a potentially harmful immune response. Survival may instead hinge on managing infection through alternative means. The variation in potential infection management mechanisms may have prevented any one gene ontology term from being enriched in our LOLA analysis. Effective conservation actions can help to prevent further extinctions of Hawaiian honeycreepers as disease‐free habitat shrinks. Actions that include the implementation of mosquito control measures and continued support of captive breeding programs for the most imperiled species.

## Author Contributions

**John H. Neddermeyer:** conceptualization (equal), data curation (equal), formal analysis (equal), methodology (equal), visualization (equal), writing – original draft (equal), writing – review and editing (equal). **Loren Cassin‐Sackett:** conceptualization (equal), funding acquisition (equal), validation (equal), writing – review and editing (equal). **Eben H. Paxton:** conceptualization (equal), funding acquisition (equal), investigation (equal), writing – review and editing (equal). **Michael G. Campana:** conceptualization (equal), funding acquisition (equal), writing – review and editing (equal). **Robert C. Fleischer:** conceptualization (equal), funding acquisition (equal), writing – review and editing (equal). **Jeffrey T. Foster:** conceptualization (equal), funding acquisition (equal), resources (equal), supervision (equal), writing – review and editing (equal).

## Funding

This work was supported by the Smithsonian Scholarly Studies program. Directorate for Biological Sciences, DEB‐2001213.

## Conflicts of Interest

The authors declare no conflicts of interest.

## Supporting information


**Figure S1:** PCA prefiltering contaminated reads from samples 2591–08576, 2591–08542, and 2591–08536.
**Figure S2:** PCA post‐filtering contaminated reads, samples 2591–08542 and 2591–08536 match expectations and retain enough sequence information to be carried forward in subsequent analyses, whereas 2591–08576 was removed because of sample likely not being from Oʻahu ʻamakihi.
**Figure S3:** Species tree phylogeny of the ʻamakihi clade. Phylogeny constructed using weighted‐ASTRAL from 7267 BUSCO gene trees. Posterior probability support for all nodes was one. Node labels represent Cactus reconstructed ancestral sequences. Ancestral sequence Anc39 was used to identify the ancestral allele for polymorphic sites associated with avian malaria infection.
**Figure S4:** Genome‐wide heterozygosity by avian malaria qPCR result. Each point represents the genome‐wide heterozygosity for a single sample. Distributions are not statistically significant from one another (Wilcoxon Rank Sum Test, p > 0.05).
**Figure S5:** ADMIXTURE cross‐validation error across K values 2–10.
**Figure S6:** Oʻahu ʻamakihi sampling locations. Pie charts show ADMIXTURE results where K = 2. Colors in pie charts represent the fraction of ʻamakihi ancestry at each site that can be attributed to one of two modeled ancestral populations.
**Figure S7:** Linear Mixed Model Genome‐wide Manhattan Plot of single nucleotide polymorphisms (SNPs) tested for association with avian malaria infection status. Scaffolds were filtered to those that had annotation information. Horizontal blue line represents the top 0.1% of ‐log10 (3.42 e−31) from the Genome‐wide Association Study. Green points are SNPs with ‐log10 (p‐value) ≥ 30.5.
**Figure S8:** Linear Model Genome‐wide Manhattan Plot of single nucleotide polymorphisms (SNPs) tested for association with avian malaria infection status. Scaffolds were filtered to those that had annotation information. Horizontal red line represents 0.30 q‐value threshold ‐log10 (4.64 e−6) from Genome‐wide Association Study. Significant SNPs are highlighted in green.
**Figure S9:** Q‐Q plot of the Gemma Linear Mixed Model.
**Figure S10:** Q‐Q plot of the Gemma Linear Model.
**Figure S11:** Annotated avian malaria infection‐associated genotypes found within genes on the basis of the top 0.1% of significant variants from GEMMA Linear Mixed Model, significant variants from GEMMA Linear Model, and pairwise FST comparisons between infected and uninfected individuals. Points show cycle threshold values. Cycle threshold values of 40 represent uninfected individuals. Panels represent a gene or locus associated with variants. Note variants annotated to TADA3 and ENSTGUG00000028750 were also annotated to a transcription start site for a locus described in Table 2.
**Figure S12:** Transcription start site (TSS) annotated avian malaria infection‐associated genotypes on the basis of the top 0.1% of significant variants from GEMMA Linear Mixed Model, and pairwise FST comparisons between infected and uninfected individuals. Points show cycle threshold values. Cycle threshold values of 40 represent uninfected individuals. Panels represent gene or locus names associated with TSS variants. Variants were annotated to a TSS if the variant fell within 5 kb upstream or downstream of a TSS. Panel (a) represents Ensembl IDs ENSTGUG00000025918, ENSTGUG00000007721, and ENSTGUG00000025413, and panel (b) represents Ensembl IDs ENSTGUG00000027186, ENSTGUG00000029360, and ENSTGUG00000027571.
**Table S1:** Association study samples and metadata.
**Table S2:** 10X linked‐read Oʻahu ʻamakihi de novo genome assembly statistics.
**Table S3:** BLASTn results for significant SNPs that were unable to be annotated to either putative regulatory regions or within genes.
**Table S4:** Single Nucleotide Polymorphism Proportion Test Results.
**Table S5:** Reconstructed ancestral allele for avian malaria prevalence‐associated single nucleotide polymorphisms.

## Data Availability

All raw sequence data and reference assemblies can be found on under NCBI BioProject accession number PRJNA1291764. All code used in the analysis can be found on GitHub page: https://github.com/John‐Neddermeyer/avian‐malaria‐associated‐genotypes‐support‐infection‐resistance‐in‐oahu‐amakihi and on Zendo DOI:10.5281/zenodo.19433027.
