# Supplementary material for: Evolution of Resistance to Avian Malaria Infection in an Endemic Hawaiian Honeycreeper
Source: Ecol Evol. 2026 May 8;16(5):e73550. doi: 10.1002/ece3.73550 (PMC13156472; doi:10.1002/ece3.73550)
Supplement: Supplementary file 1 — Figure S1: PCA prefiltering contaminated reads from samples 2591–08576, 2591–08542, and 2591–08536. Figure S2: PCA post‐filtering contaminated reads, samples 2591–08542 and 2591–08536 match expectations and retain enough sequence information to be carried forward in subsequent analyses, whereas 2591–08576 was removed because of sample likely not being from Oʻahu ʻamakihi. Figure S3: Species tree phylogeny of the ʻamakihi clade. Phylogeny constructed using weighted‐ASTRAL from 7267 BUSCO gene trees. Posterior probability support for all nodes was one. Node labels represent Cactus reconstructed ancestral sequences. Ancestral sequence Anc39 was used to identify the ancestral allele for polymorphic sites associated with avian malaria infection. Figure S4: Genome‐wide heterozygosity by avian malaria qPCR result. Each point represents the genome‐wide heterozygosity for a single sample. Distributions are not statistically significant from one another (Wilcoxon Rank Sum Test, p > 0.05). Figure S5: ADMIXTURE cross‐validation error across K values 2–10. Figure S6: Oʻahu ʻamakihi sampling locations. Pie charts show ADMIXTURE results where K = 2. Colors in pie charts represent the fraction of ʻamakihi ancestry at each site that can be attributed to one of two modeled ancestral populations. Figure S7: Linear Mixed Model Genome‐wide Manhattan Plot of single nucleotide polymorphisms (SNPs) tested for association with avian malaria infection status. Scaffolds were filtered to those that had annotation information. Horizontal blue line represents the top 0.1% of ‐log10 (3.42 e−31) from the Genome‐wide Association Study. Green points are SNPs with ‐log10 (p‐value) ≥ 30.5. Figure S8: Linear Model Genome‐wide Manhattan Plot of single nucleotide polymorphisms (SNPs) tested for association with avian malaria infection status. Scaffolds were filtered to those that had annotation information. Horizontal red line represents 0.30 q‐value threshold ‐log10 (4.64 e−6) from Genome‐wide Associati [file ECE3-16-e73550-s001.zip › supplemental figures and tables.docx]

**Supplemental Figures**

**
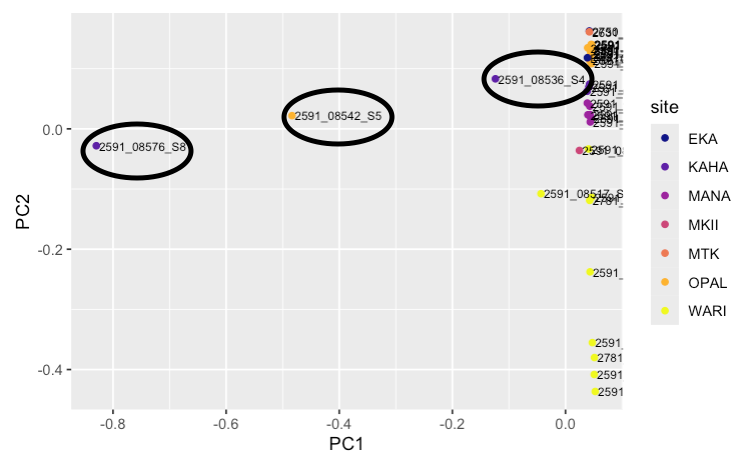
**

Supplemental Figure 1: PCA prefiltering contaminated reads from samples 2591-08576, 2591-08542 and 2591-08536.


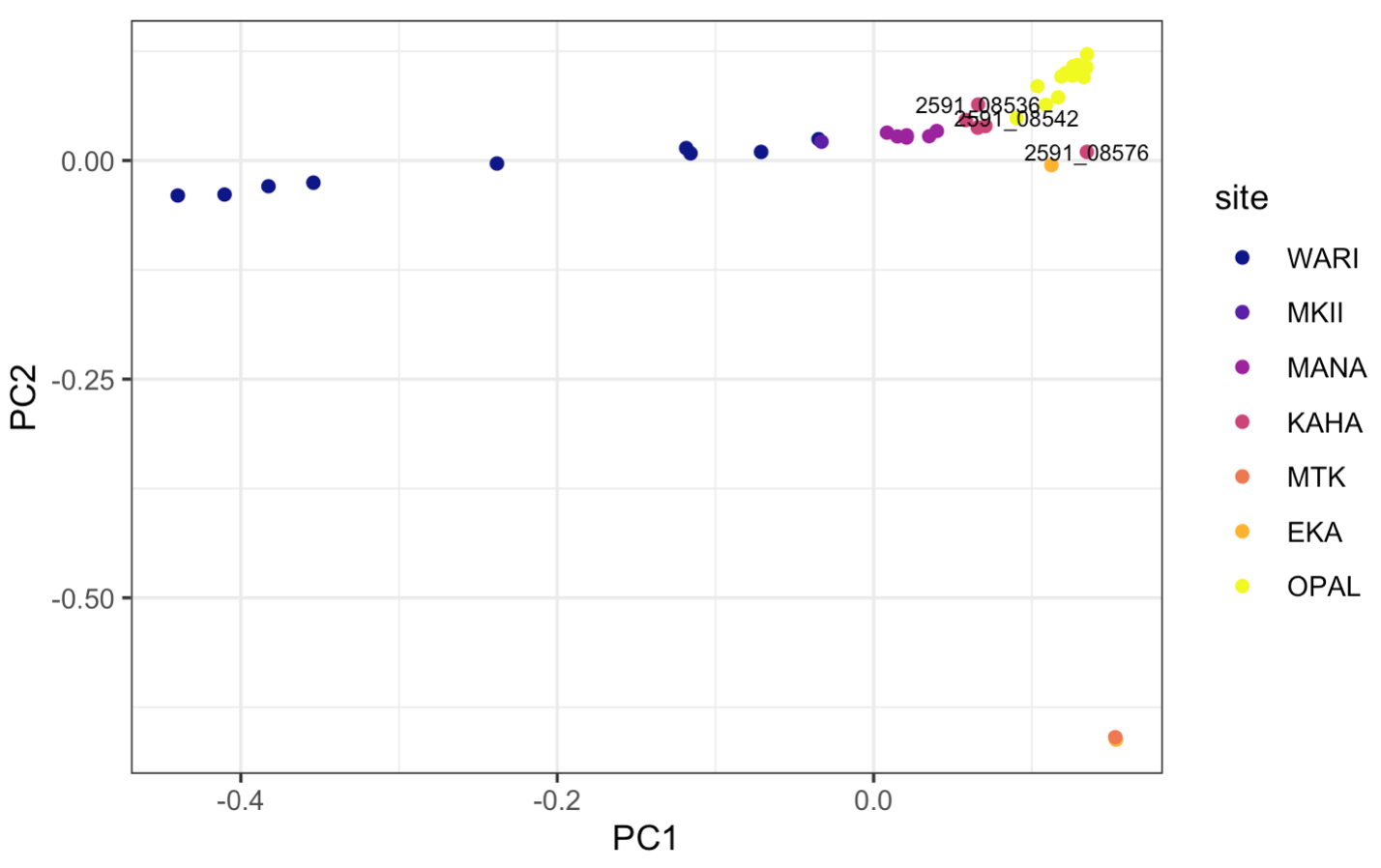


Supplemental Figure 2: PCA post-filtering contaminated reads, samples 2591-08542 and 2591-08536 match expectations and retain enough sequence information to be carried forward in subsequent analyses, while 2591-08576 was removed due to sample likely not being from Oʻahu ʻamakihi.


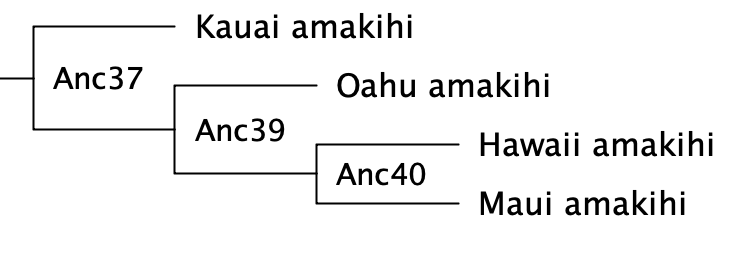


Supplemental Figure 3: Species tree phylogeny of the ʻamakihi clade. Phylogeny constructed using weighted-ASTRAL from 7,267 BUSCO gene trees. Posterior probability support for all nodes was one. Node labels represent Cactus reconstructed ancestral sequences. Ancestral sequence Anc39 was used to identify the ancestral allele for polymorphic sites associated with avian malaria infection.


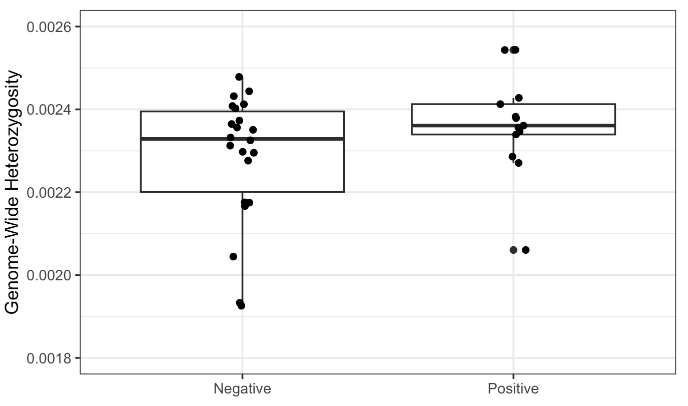


Supplemental Figure 4. Genome-wide heterozygosity by avian malaria qPCR result. Each point represents the genome wide heterozygosity for a single sample. Distributions are not statistically significant from one another (Wilcoxon Rank Sum Test, p > 0.05).


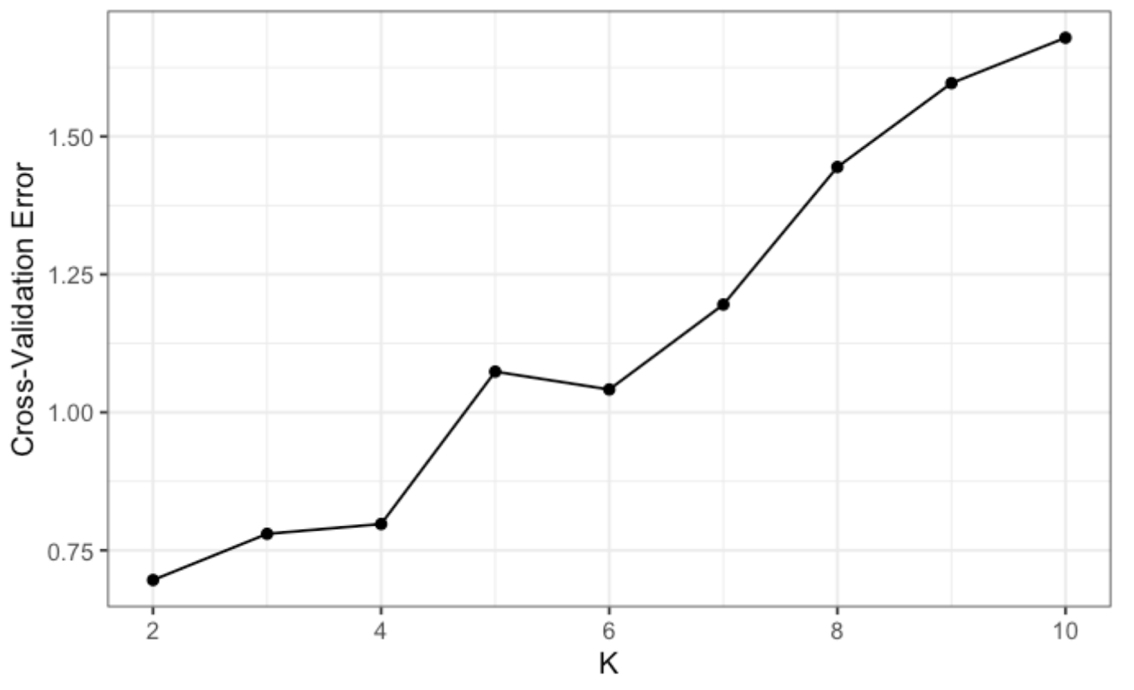


Supplemental Figure 5. ADMIXTURE cross-validation error across K values 2–10.

**
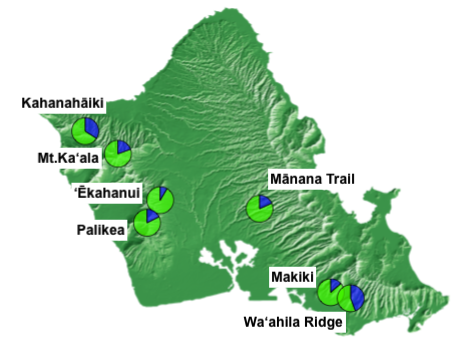
**

Supplemental Figure 6. Oʻahu ʻamakihi sampling locations. Pie charts show ADMIXTURE results where K=2. Colors in pie charts represent the fraction of ʻamakihi ancestry at each site that can be attributed to one of two modeled ancestral populations.


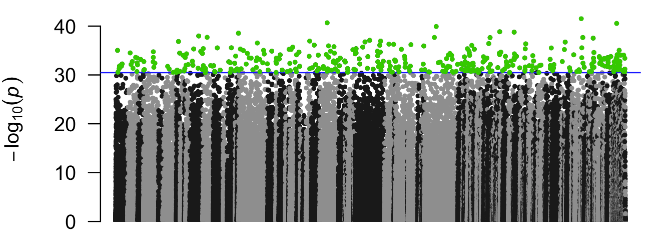
Supplemental Figure 7. Linear Mixed Model Genome-wide Manhattan Plot of Single Nucleotide Polymorphisms (SNPs) tested for association with avian malaria infection status. Scaffolds were filtered to those that had annotation information. Horizontal blue line represents top 0.1% of -log_10_(3.42 e^-31^) from Genome-wide Association Study. Green points are SNPs with -log_10_(p-value) ≥ 30.5.


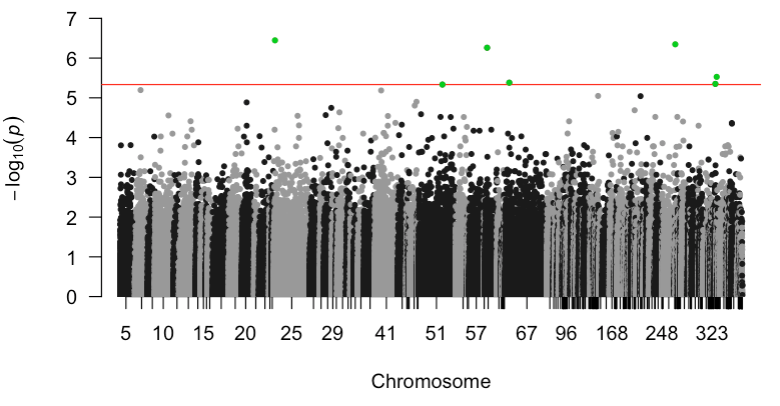


Supplemental Figure 8. Linear Model Genome-wide Manhattan Plot of Single Nucleotide Polymorphisms (SNPs) tested for association with avian malaria infection status. Scaffolds were filtered to those that had annotation information. Horizontal red line represents 0.30 q-value threshold -log_10_(4.64 e^-6^) from Genome-wide Association Study. Significant SNPs are highlighted in green.


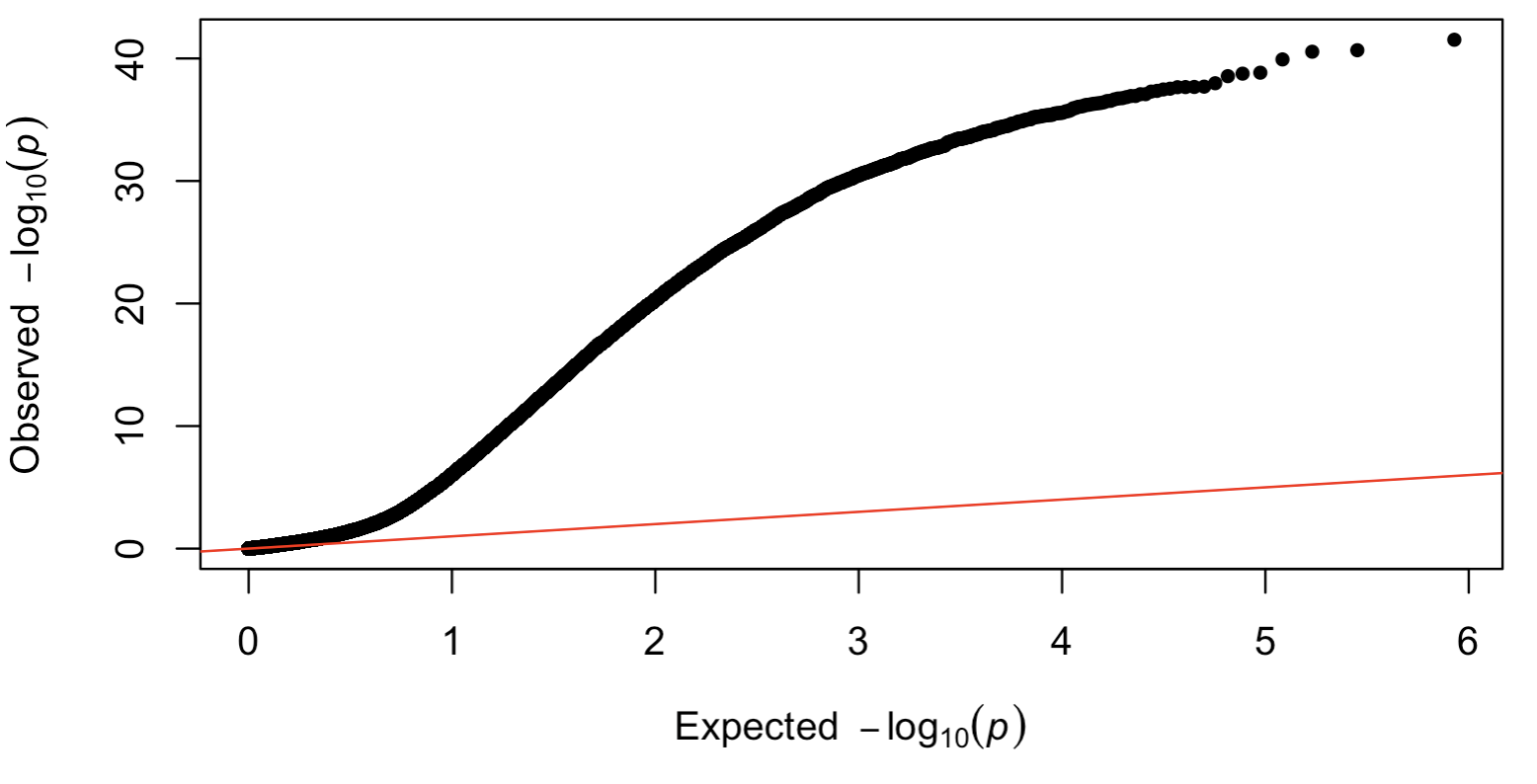
 Supplemental Figure 9. Q-Q plot of Gemma Linear Mixed Model.


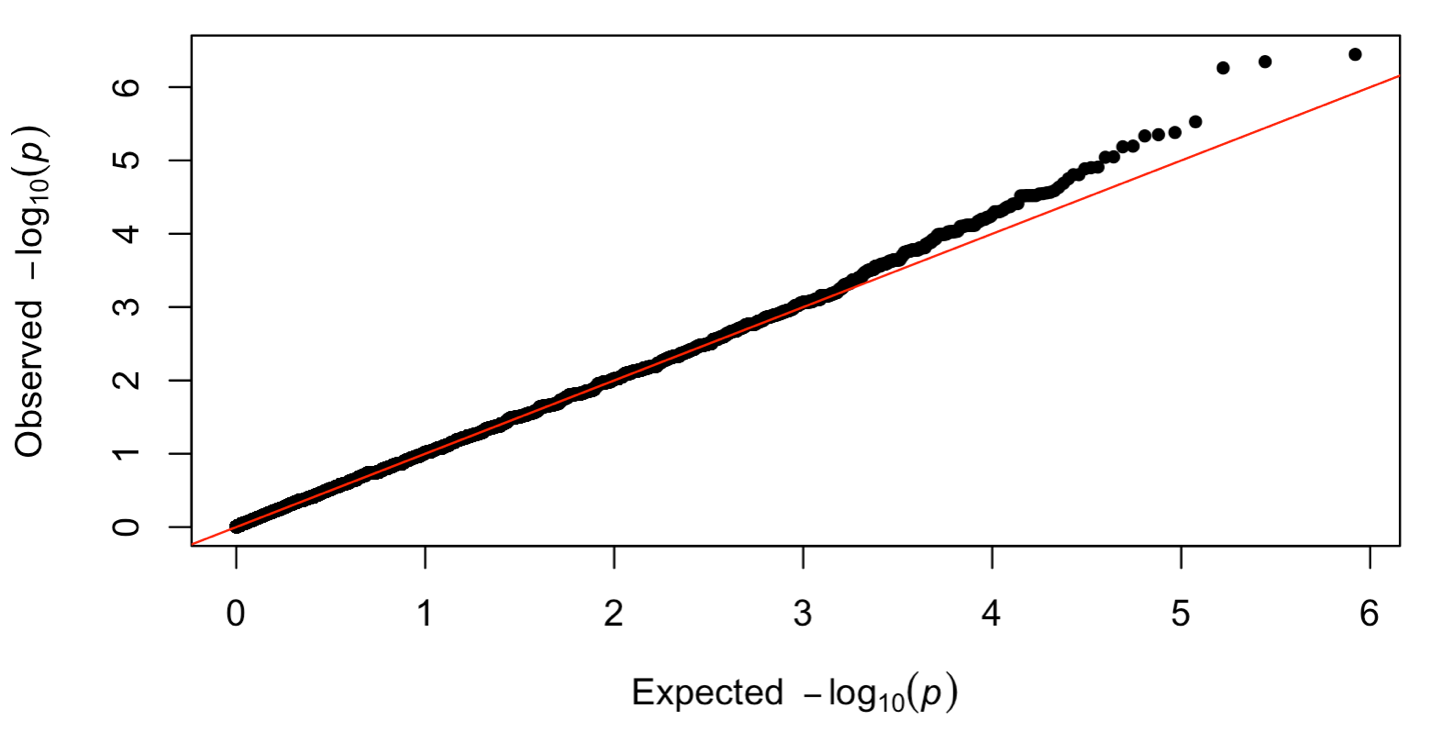


Supplemental Figure 10. Q-Q plot of Gemma Linear Model.


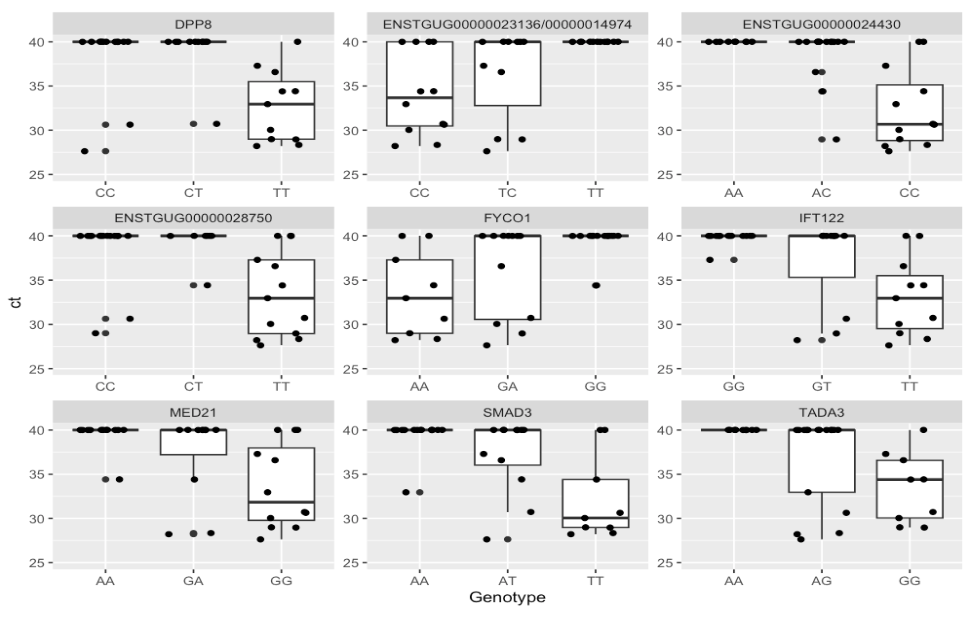


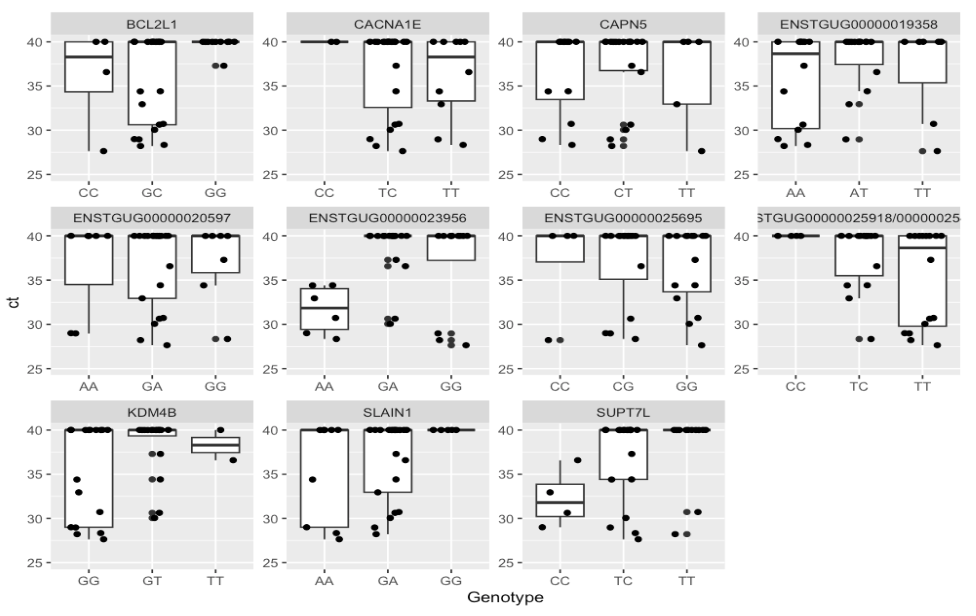


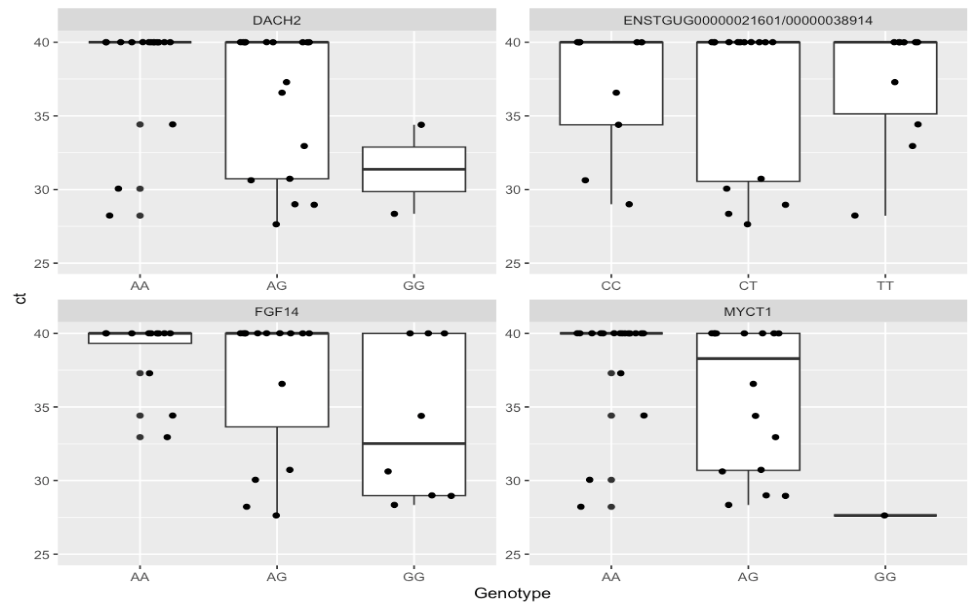


Supplemental Figure 11. Annotated avian malaria infection associated genotypes found within genes based on the top 0.1% of significant variants from GEMMA Linear Mixed Model, significant variants from GEMMA Linear Model, and pairwise FST comparisons between infected and uninfected individuals. Points show cycle threshold values. Cycle threshold values of 40 represent uninfected individuals. Panels represent gene or locus associated with variants. Note variants annotated to TADA3 and ENSTGUG00000028750 were also annotated to a transcription start site for a locus described in Table 2.


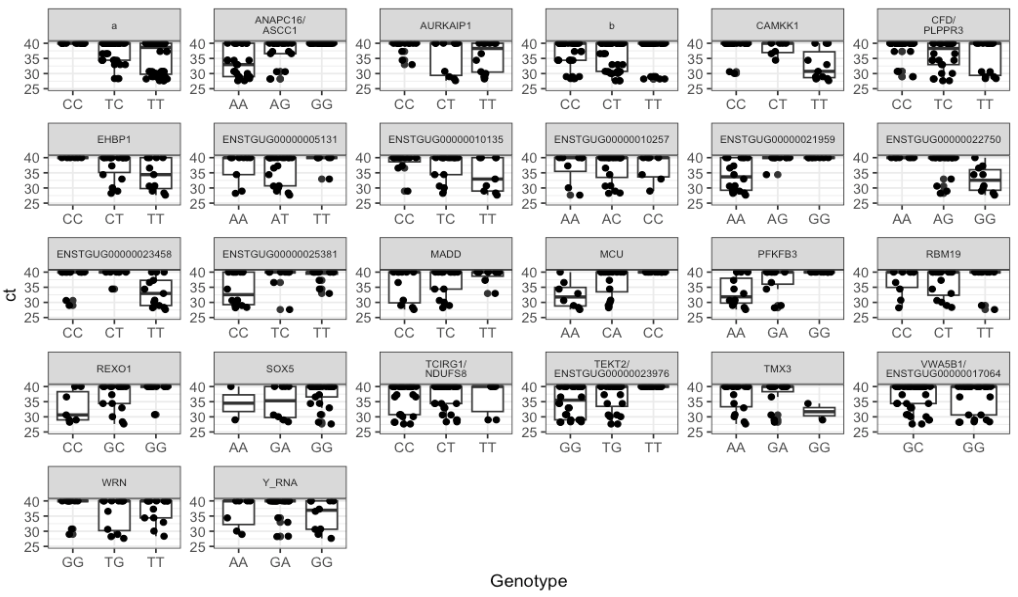
 Supplemental Figure 12. Transcription start site (TSS) annotated avian malaria infection associated genotypes based on the top 0.1% of significant variants from GEMMA Linear Mixed Model, and pairwise F_ST_ comparisons between infected and uninfected individuals. Points show cycle threshold values. Cycle threshold values of 40 represent uninfected individuals. Panels represent gene or locus name associated with TSS variants. Variants were annotated to a TSS if the variant fell within 5 kb upstream or downstream of a TSS. Panel (a) represents Ensembl IDs ENSTGUG00000025918, ENSTGUG00000007721 and ENSTGUG00000025413, and panel (b) represents Ensembl IDs ENSTGUG00000027186, ENSTGUG00000029360 and ENSTGUG00000027571.

**Supplemental Tables**

Supplemental Table 1. Association study samples and metadata.

| Band Number | Mean Sequencing Depth | %Reference Genome Coverage | Sample Site | Avian Malaria Detection | Sample Collection Date |
| --- | --- | --- | --- | --- | --- |
| 2750_36797 | 13.62 | 96.4 | ʻĒkahanui | Negative | 8/28/16 |
| 2631_31182 | 4.91 | 87.9 | ʻĒkahanui | Negative | 8/28/16 |
| 2591_08540 | 19.05 | 96.4 | Kahanahāiki | Negative | 4/27/21 |
| 2591_08563 | 10.66 | 96.1 | Kahanahāiki | Positive | 4/30/21 |
| 2591_08536 | 7.37 | 91.5 | Kahanahāiki | Positive | 4/27/21 |
| 2591_08574 | 9.48 | 95.9 | Kahanahāiki | Positive | 5/21/21 |
| 2591_08572 | 10.45 | 96.0 | Mānana Trail | Positive | 5/19/21 |
| 2591_08581 | 9.96 | 96.0 | Mānana Trail | Positive | 5/24/21 |
| 2591_08583 | 17.17 | 96.3 | Mānana Trail | Positive | 5/24/21 |
| 2591_08584 | 12.93 | 96.2 | Mānana Trail | Positive | 5/25/21 |
| 2591_08579 | 8.68 | 95.7 | Mānana Trail | Positive | 5/24/21 |
| 2591_08580 | 4.82 | 93.5 | Mānana Trail | Positive | 5/24/21 |
| 2591_08524 | 19.30 | 96.4 | Makiki | Negative | 5/25/21 |
| 2631_31193 | 63.52 | 96.7 | Mt. Kaʻala | Negative | 1/4/16 |
| 2591_08543 | 18.88 | 96.3 | Palikea | Negative | 4/28/21 |
| 2591_08546 | 21.20 | 96.4 | Palikea | Negative | 4/28/21 |
| 2591_08548 | 13.98 | 96.2 | Palikea | Negative | 4/28/21 |
| 2591_08552 | 9.78 | 95.9 | Palikea | Negative | 4/29/21 |
| 2591_08555 | 23.70 | 96.4 | Palikea | Negative | 4/29/21 |
| 2591_08559 | 7.08 | 95.3 | Palikea | Negative | 4/29/21 |
| 2591_08541 | 15.22 | 96.2 | Palikea | Negative | 4/28/21 |
| 2591_08544 | 15.64 | 96.3 | Palikea | Negative | 4/28/21 |
| 2591_08560 | 13.97 | 96.2 | Palikea | Negative | 4/29/21 |
| 2591_08562 | 19.17 | 96.4 | Palikea | Negative | 4/29/21 |
| 2591_08542 | 7.41 | 94.6 | Palikea | Negative | 4/28/21 |
| 2591_08556 | 12.27 | 96.1 | Palikea | Negative | 4/29/21 |
| 2591_08516 | 13.92 | 96.3 | Waʻahila Ridge | Negative | 5/23/21 |
| 2591_08517 | 9.82 | 95.8 | Waʻahila Ridge | Positive | 5/24/21 |
| 2591_08518 | 16.57 | 96.4 | Waʻahila Ridge | Negative | 3/24/21 |
| 2591_08520 | 15.76 | 96.2 | Waʻahila Ridge | Positive | 5/24/21 |
| 2591_08521 | 14.71 | 96.2 | Waʻahila Ridge | Negative | 5/24/21 |
| 2591_08523 | 15.70 | 96.3 | Waʻahila Ridge | Negative | 5/24/21 |
| 2591_08522 | 15.72 | 96.2 | Waʻahila Ridge | Positive | 3/24/21 |
| 2781_72510 | 11.08 | 96.1 | Waʻahila Ridge | Positive | 3/24/21 |
| 2781_72516 | 12.43 | 96.2 | Waʻahila Ridge | Negative | 5/23/21 |

Supplemental Table 2. 10X linked-read Oʻahu ʻamakihi *de novo* genome assembly statistics.

| Species | Assembly length (GB) | Scaffold Number | Longest scaffold (MB) | Scaffold N50 (MB) | Contig N50 (KB) | % Complete BUSCOs | % N |
| --- | --- | --- | --- | --- | --- | --- | --- |
| Oʻahu ʻamakihi (*Chlorodrepanis flava*) | 1.03 | 13551 | 61.78 | 12.99 | 117.2 | 95 | 3.2 |

Supplemental Table 3. BLASTn results for significant SNPs that were unable to be annotated to either putative regulatory regions or within genes.

| NCBI Accession Number | Locus Description | SNP |
| --- | --- | --- |
| XM_066561177.1 | dehydrogenase/reductase 7B (DHRS7B) | simple_201:4037054 |
| XM_059864410.1 | Rho GDP dissociation inhibitor alpha (ARHGDIA) | simple_271:1188565 |
| XM_050983043.1 | TIA1 cytotoxic granule associated RNA binding protein (TIA1) | simple_3314:1228 |
| XM_059866836.1 | spermidine synthase (SRM) | simple_343:187429 |
| XM_009093509.4 | pappalysin 1 (PAPPA) | simple_400:5039257 |
| XM_059845157.1 | heparan sulfate-glucosamine 3-sulfotransferase 1 (HS3ST1) | simple_41:23218152 |
| XM_059849792.1 | Rho GTPase activating protein 11A (ARHGAP11A) | simple_42:10610281 |
| XR_003945237.2 | uncharacterized LOC115484572 | simple_5:6802670 |
| XR_009488153.1 | uncharacterized LOC132333403 | simple_7077:1196 |
